# Expression Landscape and Circadian Regulation of lncRNAs in the Kidney

**DOI:** 10.1111/apha.70273

**Published:** 2026-07-03

**Authors:** Leonore Wigger, Fanny Durussel, Muriel Auberson, Dmitri Firsov, Yohan Bignon

**Affiliations:** ^1^ Department of Biomedical Sciences University of Lausanne Lausanne Switzerland; ^2^ Vital‐IT, Swiss Institute of Bioinformatics Lausanne Switzerland

**Keywords:** cell‐type specificity, circadian clock, circadian rhythm, dryR, Gini coefficient, long noncoding RNA, renal tubule, single‐nucleus sequencing

## Abstract

Recent multi‐omics work shows that the diurnal rhythmicity of kidney function is associated with circadian oscillations in renal mRNA, protein and metabolite abundances. Yet circadian regulation of renal lncRNAs, which are emerging as important modulators of diverse (patho)physiological processes, remains largely unexplored. Here, we provide a comprehensive characterization of renal lncRNA expression across cell types and circadian time points using bulk RNA‐Seq and single‐nucleus RNA‐Seq (snRNA‐seq) using kidney samples from male mice. Although the majority of lncRNAs are lowly expressed and only a fraction are annotated, their profiles sufficed to distinguish nearly all renal cell types. Compared with mRNAs, lncRNAs were more cell‐type specific and an inverse correlation between cell‐type specificity and expression level was evident. To assess the role of the circadian clock, we used mice lacking the core‐clock regulator BMAL1 in renal tubules. Approximately one‐sixth of lncRNAs and one‐third of mRNAs were rhythmic. In contrast with mRNAs, lncRNAs exhibited an asymmetric temporal distribution between night and day, with peak expression occurring preferentially during the inactive phase of the mice. *Bmal1* deletion partially disrupted rhythmicity in both biotypes and altered overall expression levels; lncRNAs were predominantly upregulated. These findings uncover the diversity of renal lncRNAs, their cellular distribution and their circadian regulation in male mice.

## 1 Introduction

Long noncoding RNAs (lncRNAs) are defined as noncoding RNAs longer than 200 nucleotides. This broad definition, based solely on an arbitrary length cutoff, is currently under debate because it groups together noncoding RNAs of diverse origins, properties, and functions. Hence, lncRNAs can modulate many key aspects of cellular biology, such as chromatin architecture and DNA transcription, splicing, localization, availability or stability of RNAs and translation, scaffolding, or localization of proteins, through direct or indirect interactions with DNA (promoters, enhancers, etc.), RNAs (mRNAs, circRNAs, miRNAs, etc.), and proteins [1, 2, 3]. In general, lncRNAs are classified into four groups based on their expression patterns and mechanisms of action: “Decoy lncRNAs” regulate transcription factor binding to DNA, RNA–RNA interactions, RNA stability or degradation, and protein translation exclusively through the binding and titration of target RNAs or proteins; for example, some lncRNAs act as miRNA sponges by binding and sequestering miRNAs from their target mRNAs. “Guide lncRNAs” bind nucleic acids or proteins and direct them to specific targets (chromatin, partners, or regulatory complexes). “Scaffold lncRNAs” interact with multiple partners to form protein or ribonucleoprotein complexes involved in regulating various functions, including transcription or chromatin structure. Finally, “signal lncRNAs”, whose spatio‐temporal expression is controlled by external stimuli, contribute to intracellular signaling [1, 4, 5, 6].

It is now widely accepted that lncRNAs are, on average, expressed at severalfold lower levels than mRNAs. Moreover, lncRNAs tend to display more restricted expression patterns than mRNAs; that is, a larger proportion appears to be highly specific to particular cell types, tissues, organs, or taxonomic groups [7, 8, 9, 10, 11]. The expression of many lncRNAs has been shown to be strongly modulated during development, with signal lncRNAs contributing to organ formation through their tissue‐specific or cell‐specific expression at given developmental stages [12], and they can also be affected by aging [13], similar to what is observed for many mRNAs. Moreover, lncRNAs can be regulated by changes in cell state, for example, during cell differentiation or under endoplasmic reticulum stress [4, 14, 15]. However, only a handful of studies have addressed the rhythmicity of lncRNAs across the circadian cycle and its relationship to the circadian clock. Recently, Miao et al. [16] used machine learning models to predict rhythmic transcriptional patterns, and their results suggested that regulatory mechanisms governing rhythmic transcription may differ between mRNAs and lncRNAs, a possibility that calls for experimental validation.

Research on the role of lncRNAs in renal (patho)physiology is still at a nascent stage. Studies have revealed that in the kidney, the expression of many lncRNAs is modulated during development [12] and in the course of renal disorders [17, 18, 19, 20, 21]. A few dozen renal lncRNAs have been shown to be mechanistically involved in renal pathogenesis or disease progression, mostly by interfering with pathways related to inflammation, oxidative stress, fibrosis, or apoptosis through the regulation of miRNAs or NF‐κB activity [22, 23, 24]. For instance, increased renal expression of the lncRNAs PVT1 or XIST induces extracellular matrix production or apoptosis, respectively, in kidney diseases of multiple etiologies [25]. However, the distribution of lncRNAs across renal cell types remains largely unknown.

Here, we chart the expression landscape of Ensembl‐annotated (www.ensembl.org) lncRNAs in the mouse kidney, quantifying their abundance, cell‐type specificity and distribution among cell types. In light of our previous finding that numerous renal mRNAs exhibit robust circadian rhythms [26], we also characterize the circadian expression patterns of lncRNAs and their dependence on the tubule‐intrinsic circadian clock. In mammals, circadian rhythms are regulated by a clock mechanism, present in nearly all tissues, composed of a system of transcriptional‐translational feedback loops involving core‐clock genes such as *Clock*, *Bmal1*, *Per*1–3, *Cry*1–2, and *Npas2*. The transcription factor CLOCK (or its paralog NPAS2, which is preferentially expressed in the forebrain and certain peripheral tissues) dimerizes with BMAL1 to drive the transcription of *Per* and *Cry* genes via E‐box elements. PER and CRY proteins form complexes that inhibit CLOCK:BMAL1 (or NPAS2:BMAL1) activity, forming a negative feedback loop [27]. Auxiliary loops involving transcription factors encoded by *Rev‐erb* (*Nr1d1*, *Nr1d2*) and *Ror* (*Rora*, *Rorb*, *Rorc*) genes enhance stability and precision of core‐clock gene oscillations through D‐box and RORE response elements [28]. The core clock mechanism drives rhythmic expression of numerous circadian “output” genes, including the PAR bZip transcription factors DBP, HLF and TEF [29].

We observed that renal lncRNAs are, on average, more cell‐type specific and less abundant than mRNAs, but that these two traits are associated in both RNA classes. The lower the abundance of an RNA of either type, the more likely it is to exhibit high cell‐type specificity. We further found that renal lncRNA expression profiles in individual nuclei are sufficiently distinctive to discriminate among most renal cell types. Moreover, our study showed that many renal lncRNAs oscillate with a 24‐h period. In a transgenic mouse model in which the circadian clock mechanism is selectively disrupted in renal tubules, some lncRNAs lose their circadian rhythms, others acquire rhythmicity absent in control mice, and yet others retain their rhythms, with or without phase shifts or amplitude changes.

## 2 Results

### 2.1 Diversity and Cell‐Type Specificity of Renal lncRNAs

We analyzed three related murine kidney RNA‐seq datasets derived from male animals: one single‐nucleus dataset and two bulk datasets (see Section 4). We refer to them as single‐nucleus data (SN), deeply sequenced bulk data (DS), and circadian bulk data (Circ). Each dataset comprises kidney samples collected from two genotypes: mice whose circadian clock had been specifically disrupted in renal tubules (cKOt) through genetic invalidation of the gene *Bmal1*, a key regulatory gene of the circadian core‐clock mechanism [26, 30], and their littermates, which served as controls (Ctrl). The mice were maintained under a 12‐h light/12‐h dark cycle with ad libitum feeding for a month prior to tissue collection at different Zeitgeber Times (ZT). ZT is a standardized 24‐h time notation referring to the diurnal light–dark schedule, with ZT 0–12 corresponding to the light phase when mice are normally inactive, and ZT 12–24 to the dark phase when mice are active.

To assess lncRNA expression levels and lncRNA diversity across renal cell types, we first analyzed the SN dataset, which comprised eight kidney samples harvested from two replicate mice per genotype at two time points, ZT4 and ZT16. When using a predefined annotation, lncRNA diversity, that is, the number of distinct lncRNAs detected in a dataset, depends on multiple factors: how many features are annotated, how many are actually present in the examined samples, and how sensitive the assay is to picking up the lowly expressed ones. The Ensembl annotation used for the SN dataset, obtained from 10× Genomics (https://www.10xgenomics.com/) and based on genome assembly GRCm39.110, included 9888 lncRNAs (annotated as “lncRNA”) and 21 700 mRNAs (annotated as “protein_coding”). Of these, 7553 lncRNAs and 18 705 mRNAs had at least one read mapped to them and were thus included in the processed SN dataset. To characterize overall lncRNA expression in renal nuclei and contrast it with mRNA expression, initial analyses were performed using only the four Ctrl samples, in which 7037 lncRNAs and 17 302 mRNAs annotated in Ensembl had read counts.

**FIGURE 1:**
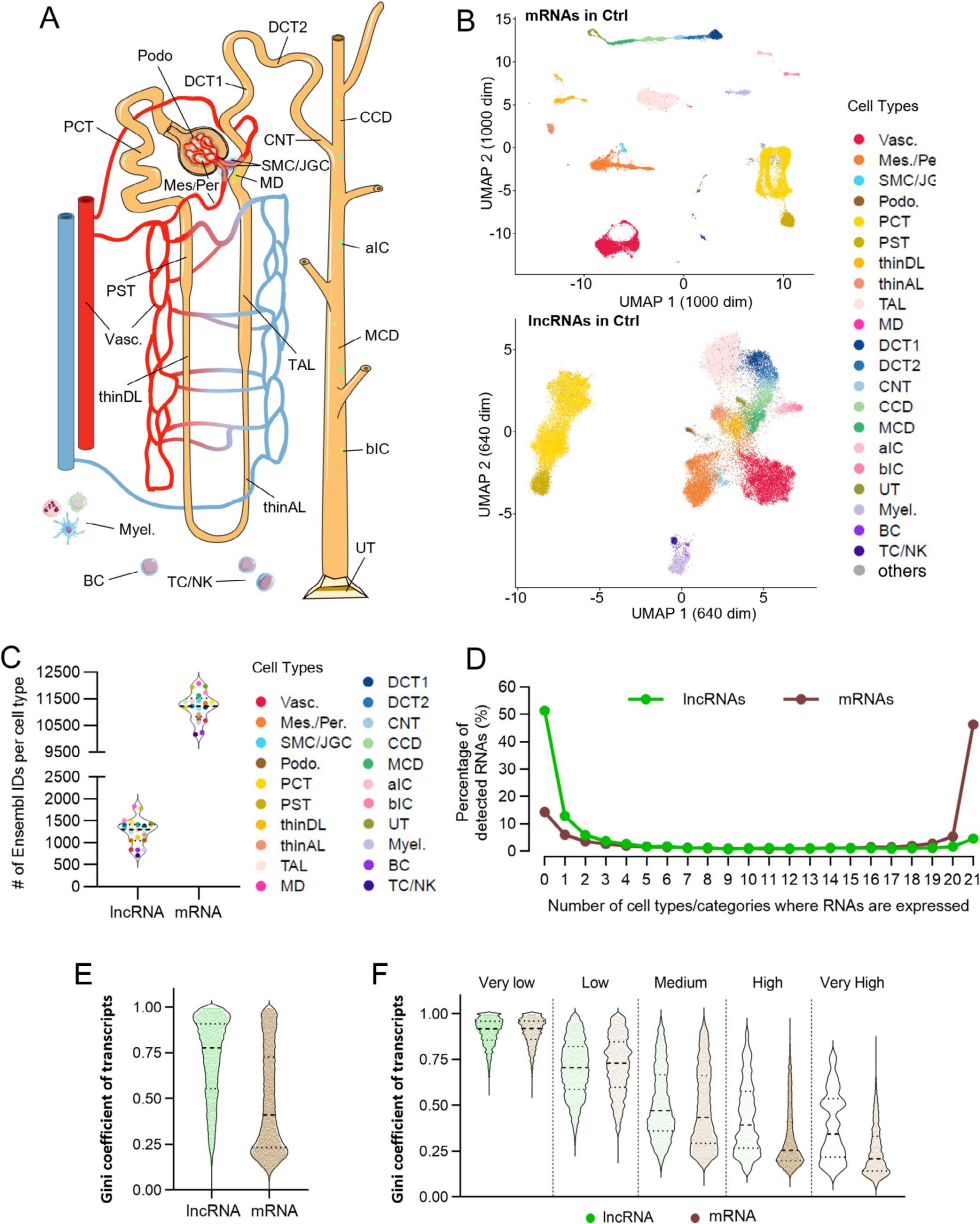
Diversity and specificity of lncRNAs in renal cells. (A) Schematic representation of a renal tubule and cell types/categories identified by single‐nucleus analysis. aIC, alpha‐intercalated cells; BC, B cells; bIC, beta‐intercalated cells; CCD, principal cells of the cortical collecting duct; CNT, connecting tubule; DCT1, distal convoluted tubule; DCT2, distal convoluted tubule; MCD, principal cells of the medullary collecting duct; MD, Macula densa; Mes/Per, mesangial cells and pericytes; Myel., myeloid cells (macrophages, neutrophils, basophils, dendritic cells); PCT, proximal convoluted tubule; Podo, podocytes; PST, proximal straight tubule; SMC/JGC, vascular smooth muscle cells and juxtaglomerular cells; TAL, thick ascending limb; TC/NK, T cells and natural killer cells; thinAL, thin ascending limb; thinDL, thin descending limb; UT, urothelium; Vasc., vascular cells. (B) UMAP generated from renal mRNAs (upper plot) or lncRNAs (lower plot) in Ctrl mice, ZT4 and ZT16 (*N* = 4). Nuclei are colored by their attributed cell type/category. (C) Gene diversity in lncRNAs and mRNAs. Violin plots contain a data point for each of 21 cell types/categories, representing the number of distinct features (Ensembl IDs) in each cell type. Detection threshold: At least 1 count in ≥ 1% of cells of a cell type. Violin plot width is scaled by number of counts. Jitter along *x*‐axis is random. Dotted lines: Median number of Ensembl IDs per cell type. (D) Prevalence of detected mRNAs (brown line) and lncRNAs (green line) among cell types/categories. *Y*‐axis: Percentages of RNAs (Ensembl IDs). *X*‐axis: Number of cell types in which RNAs were detected. Detection threshold: At least 1 count in ≥ 1% of cells of a cell type. RNAs that did not pass the threshold in any cell type were excluded before percentage calculation. (E) Cell‐type specificity, measured by Gini coefficient, of all lncRNAs and mRNAs in Ctrl. No low‐abundance filtering was applied. Dotted lines: Median and first/last quartiles. (F) Cell‐type specificity, measured by Gini coefficient, of all lncRNAs and mRNAs, stratified by averaged expression level (A‐value: Means of pseudo‐bulk counts, normalized, log2‐transformed and averaged across 4 Ctrl mice). Very low: A < 3; Low: 3 ≤ A < 6, Medium: 6 ≤ A < 9, High: 9 ≤ A < 12, Very High: 12 ≤ A < 15. Outlier features (7 lncRNAs, 87 mRNAs) with A ≥ 15 were excluded. No low‐abundance filtering was applied. Dotted lines: Median and first/last quartiles.

Using unsupervised clustering analysis and UMAP projection on mRNA features exclusively, we were able to assign 97.7% of the 81 346 QC‐filtered nuclei to 21 cell types (or cell categories) identified manually by canonical marker genes (Figure 1A, Figure S1A; Table S1; see Data S2). The cell‐type proportions obtained were in line with previously published renal single‐cell data [31, 32]. The resulting cell‐type annotations from this initial analysis, based on mRNAs from all eight SN samples, were retained for all subsequent analyses performed on subsets of samples. The top of Figure 1B displays a UMAP projection of nuclei from the four Ctrl samples derived from their mRNA expression profiles, and below it, a corresponding projection based exclusively on lncRNA expression. As expected, the UMAP from mRNA shows well‐separated 2D structures that correspond closely to the identified cell types. Intriguingly, the UMAP from lncRNAs also groups cells according to their assigned cell type, even though lncRNAs were excluded from the dataset for the purpose of cell type labeling. Three major 2D structures are distinguishable: the first one contains epithelial cells of straight and convoluted segments of the proximal tubule; the second one corresponds to immune cells; and the last one brings together epithelial cells from the loop of Henle, the distal tubule segments and the collecting duct as well as glomerular cells and vascular cells. It is split into substructures that correspond well to assigned cell types. An independent clustering of nuclei from Ctrl mice based solely on lncRNA expression produced 40 clusters that also show strong visual correspondence with cell types (Figure S2B; compare with cell‐type coloring in the bottom part of Figure 1B). Only a few, closely related cell types were not distinguished from one another when relying exclusively on lncRNA data: DCT2 and CNT, CCD and MCD, as well as aIC and bIC fall in shared clusters; SMC/JGC are clustered with some Mes./Per.; MD neither forms a cluster nor displays as a cohesive point cloud in the UMAP and is visually merged with TAL cells. These results show that lncRNAs are sufficient to delineate most renal cells on a UMAP and to produce clusters that correspond to cell types identified using usual mRNA markers.

As lncRNAs are generally considered more cell‐type specific than mRNAs, we analyzed the number of lncRNAs and mRNAs detected in each identified renal cell type and vice versa. Considering only RNAs present in at least 1% of cells within each cell type, we found that each renal cell type expresses ~500–2000 lncRNAs corresponding to ~7%–28% of all detected lncRNAs, as well as 10 000–12 000 mRNAs corresponding to 58%–69% of all mRNAs (Figure 1C). As depicted in Figure S2C, lncRNA diversity and mRNA diversity are largely proportional for most cell types, with Macula Densa (MD) and Proximal Straight Tubule (PST) cells showing the highest diversity levels and immune cells showing low diversity levels. Moreover, many lncRNAs do not pass the 1% cell‐proportion threshold in any cell type at all or are found in exactly one or in a few cell types (mean ± SEM: 3.8 ± 0.08, median: 0), while a large fraction of mRNAs are expressed in all or nearly all cell types (mean ± SEM: 13.6 ± 0.07, median: 20) (Figure 1D; see Supporting data values Data S1). Beyond these observations, there is currently no well‐established definition nor widely used measure for cell‐type specificity of an RNA feature in single‐cell data. The Gini coefficient, a measure originally developed in economics to quantify income inequality, has been adopted for this purpose in several single‐cell studies, with variations in its definition [33, 34, 35, 36]. In our analysis, a Gini coefficient of 1.0 indicates that all read counts for a gene are confined to a single cell type, while a value of 0.0 signifies that all cell types contain the same proportion of cells in which the gene is detected (≥ 1 read). In our kidney dataset, lncRNAs exhibit significantly higher average Gini coefficients compared to mRNAs (lncRNA mean = 0.719, mRNA mean = 0.481; Welch's *t*‐test: estimated difference = 0.238, *p* = 0.00), indicating greater overall cell‐type specificity (Figure 1E; Table S2). However, stratifying the data into five expression‐level groups reveals that all very lowly expressed features display high cell‐type specificity, without any exceptions and regardless of biotype (Figure 1F; Table S2). In the three highest expression groups (strata 3, 4, 5), lncRNAs remain more specific than mRNAs, but the magnitude of these differences is modest and substantially smaller than the differences observed between expression strata. In the highest expression category (stratum 5), no lncRNA nor mRNA is perfectly confined to a single or few cell types, and the highest Gini coefficients are far below 1.0 (lncRNAs: 0.11–0.78; mRNAs: 0.10–0.72). In contrast, restriction to a single cell type is common for the lowest expression group (Gini coefficients in lncRNAs: 0.58–1.0, mRNAs: 0.55–1.0). Since lncRNAs are disproportionately represented in the lowest expression category (Figure S2D), the global distribution of their Gini coefficients is biased upward in comparison to mRNAs. In both lncRNA and mRNA, the strength of the inverse correlation between Gini coefficient and expression level is similar (lncRNA: Pearson *r* = −0.78, mRNA: Pearson *r* = −0.80, Figure S2E). Altogether, these results suggest that, in line with the consensus, renal lncRNAs might typically be expressed in a more restricted number of cells or cell types than renal mRNAs. However, this might primarily be a function of their very low expression levels and only to a much lesser extent reflect a unique characteristic of lncRNAs independent of expression magnitude.

Transcripts with near‐exclusive expression in a single cell type (Gini coefficient ≥ 0.95) are lowly expressed, with per‐cell‐type read counts generally in the single digits. Among them, we identified 20 lncRNAs and 27 mRNAs with ≥ 5 pseudo‐bulk counts in at least two replicates that can be considered as valid hits. Thus, several lncRNAs appear to be specifically and consistently expressed in podocytes, MD, or beta‐intercalated cells (Figure S2F).

### 2.2 Circadian Time and Circadian Clock Dependence of Renal lncRNA Expression

Because lncRNA expression patterns are thought to be tightly controlled in both space and time, we wondered whether the expression levels of renal lncRNAs depend on circadian time and whether they might be controlled by the intrinsic circadian clocks in renal tubules. While the cell‐intrinsic clocks in peripheral organs such as the kidney exhibit autonomous oscillations, they are usually synchronized by systemic signals under the control of the suprachiasmatic nucleus (SCN), which ensures coherent circadian regulation across tissues [37]. In the cKOt transgenic mouse model, the clock mechanism in the renal tubules is impaired, but signaling from the central clock in the SCN continues to drive the circadian oscillations of many renal RNAs indirectly via factors extrinsic to kidney cells [26].

To verify that oscillations of core clock genes are observable in the SN data from Ctrl mice, we assessed transcript abundance and differential expression between ZT16 and ZT4 for 16 core genes of the circadian clock: *Clock, Npas2, Arntl, Per1, Per2, Cry1, Cry2, Nr1d1, Nr1d2, Dbp, Tef, Hlf, Nfil3, Rora, Rorb*, and *Rorc*. All of them passed low‐expression filtering. In the *p*‐value–ranked differential expression result list from SN pseudo‐bulk data (Table S3), seven clock genes (*Nr1d1, Cry1, Dbp, Npas2, Arntl/Bmal1, Rorc*, and *Nr1d2*, in this order) were among the top 10. Fisher's one‐tailed exact test provided strong statistical evidence for enrichment of the selected clock genes in the top 25 (*p* = 3.437^−17^). Analysis of individual cell types further confirmed the detectability of time‐dependent differential expression of clock genes in our SN data. All cell types in the cortical segments of the renal tubules (PCT, PST, tDL, TAL, DCT1, DCT2, CNT) and collecting ducts (CCD, aIC, bIC) as well as mesangial/pericyte and vascular cells (Mes/Per, Vasc) had at least one clock gene in the top three and up to eight among the top 25, with *p*‐values for enrichment computed by Fisher's test ranging from 8.7099^−21^ to 3.586^−05^ (Table S4).

**FIGURE 2:**
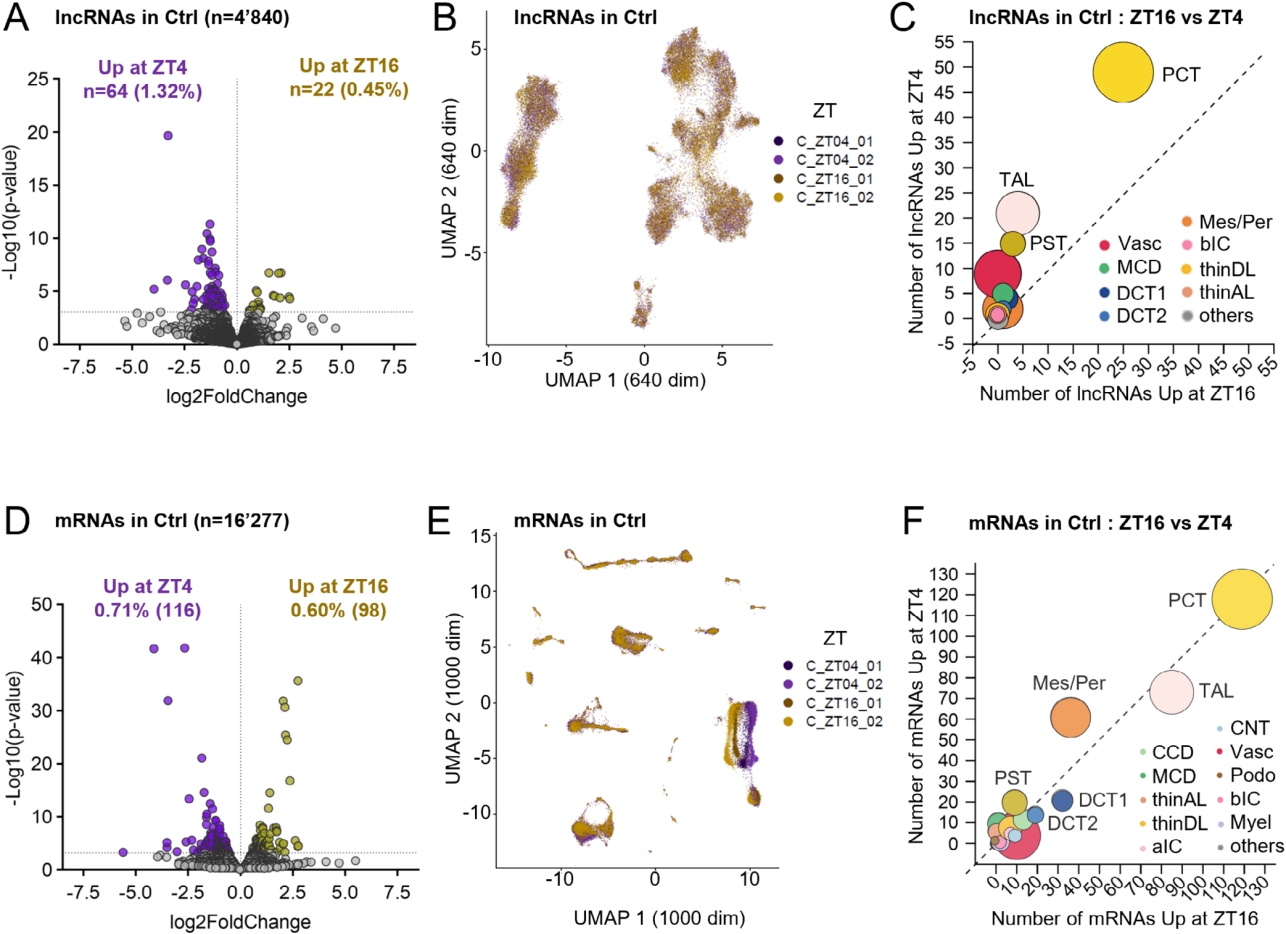
Effects of circadian time on renal lncRNA and mRNA expression in snSeq data. (A) Volcano plot of differential lncRNA expression between ZT4 and ZT16 in Ctrl mice, based on pseudo‐bulk analysis of the single‐nucleus (SN) dataset. lncRNAs with significant fold changes (adjusted *p* < 0.05, threshold shown as dotted line) are highlighted in purple (more abundant at ZT4) or yellow (more abundant at ZT16). Numbers and percentages of differentially expressed lncRNAs are indicated at the top of the plot. (B) UMAP generated from renal lncRNAs in Ctrl mice, ZT4 and ZT16. Nuclei are colored by sample (purple hues: ZT4, yellow hues: ZT16). (C) Number of lncRNAs more abundant at ZT4 (*y*‐axis) vs. more abundant at ZT16 (x‐axis) in Ctrl mice for each renal cell type (colored dots). Dot size is proportional to the number of nuclei attributed to the cell type. (D) Volcano plot of differential mRNA expression between ZT4 and ZT16 in Ctrl mice, based on pseudo‐bulk analysis of the single‐nucleus (SN) dataset. mRNAs with significant fold changes (adjusted *p* < 0.05, threshold shown as dotted line) are highlighted in purple (more abundant at ZT4) or yellow (more abundant at ZT16). Numbers and percentages of differentially expressed mRNAs are indicated at the top of the plot. (E) UMAP generated from renal lncRNAs in Ctrl mice at ZT4 and ZT16. Nuclei are colored by sample (purple hues: ZT4, yellow hues: ZT16). (F) Number of mRNAs more abundant at ZT4 (*y*‐axis) vs. more abundant at ZT16 (*x*‐axis) in Ctrl mice for each renal cell type (colored dots). Dot size is proportional to the number of nuclei attributed to the cell type.

As an initial approach to explore whether renal lncRNAs may exhibit time‐of‐day–dependent variation, we performed a differential expression analysis between ZT4 and ZT16 in control mice through a pseudo‐bulk analysis of our SN data. Figure S2 shows two UMAPs of lncRNAs and two UMAPs of mRNAs, generated separately from the ZT4 and the ZT16 time point, colored by assigned cell types. We identified 86 lncRNAs (1.77% of lncRNAs included in the SN dataset) that were differentially expressed between ZT4 and ZT16, of which 64 (1.32%) were more abundant at ZT4 and only 22 (0.45%) at ZT16 (Figure 2A). This asymmetry (~75% of time‐sensitive lncRNAs peak during the early light phase) suggests a potential phase preference or functional clustering of lncRNA activity around ZT4 (Table S5). To evaluate the distribution of differentially expressed lncRNAs among renal cell types or nephron segments, we analyzed lncRNA expression at the cell‐type level. UMAP representation showed that ZT4 and ZT16 nuclei of Ctrl mice overlapped broadly across renal cell types, with no strong time‐of‐day separation (Figure 2B). Consistent with the pseudo‐bulk analysis, we observed a clear temporal asymmetry in nearly all renal cell types, with more lncRNAs upregulated at ZT4 than at ZT16, reinforcing the idea of a time‐of‐day bias in lncRNA expression within cells. Among identified renal cell types, PCT, TAL and PST segments showed the highest numbers of differentially expressed lncRNAs between ZTs (Figure 2C; Table S6). We applied the same analysis to mRNAs and found that a similar proportion of mRNAs (~1.31%) were differentially expressed between ZT16 and ZT4 as for lncRNAs, but with a more balanced distribution between ZTs: 112 mRNAs (0.65%) exhibited higher expression at ZT4, while 105 (0.62%) were more abundant at ZT16 (Figure 2D). In the UMAP generated from mRNAs, the nuclei of PCT cells from the two time points appear clearly separated (Figure 2E), advocating for a stronger diurnal structuring of mRNA expression in this nephron segment than in other renal cell types. Correspondingly, PCT cells showed the largest number of mRNAs that were differentially expressed between time points, without the ZT4‐skewed asymmetry observed in lncRNAs (Figure 2F; Table S6).

**FIGURE 3:**
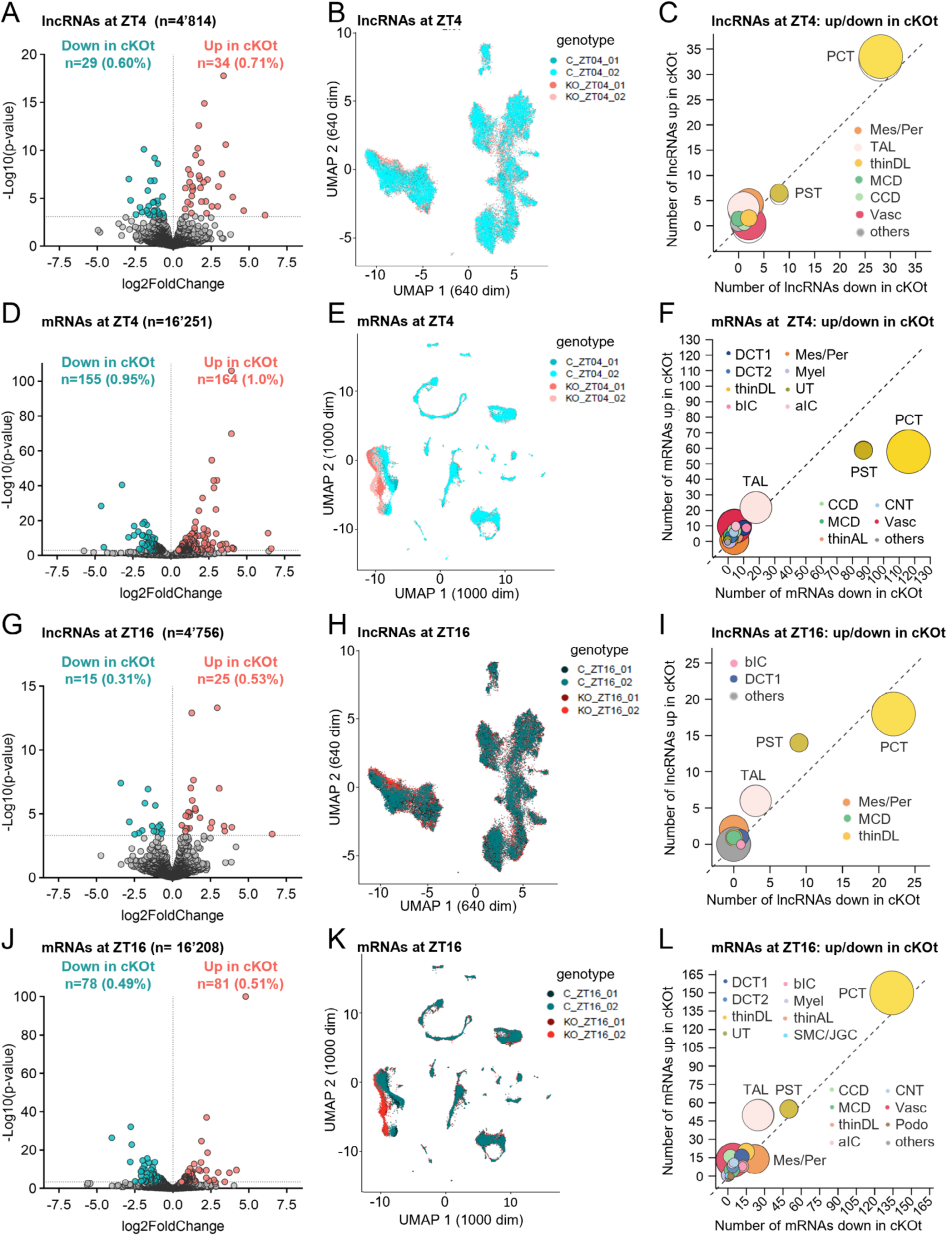
Effects of the circadian clock on renal lncRNA and mRNA expression in snSeq data. (A) Volcano plot of differential lncRNA expression between cKOt and Ctrl at ZT4, based on pseudo‐bulk analysis of the single‐nucleus (SN) dataset. lncRNAs with significant fold changes (adjusted *p* < 0.05, threshold shown as dotted line) are highlighted in blue (less abundant in cKOt) or red (more abundant in cKOt). Numbers and percentages of differentially expressed lncRNAs are indicated at the top of the plot. (B) UMAP generated from lncRNAs in Ctrl and cKOt mice, ZT4. Nuclei are colored by sample (blue hues: Ctrl, red hues: CKOt). (C) Number of lncRNAs more abundant (*y*‐axis) vs. less abundant (*x*‐axis) in cKOt than in Ctrl mice at ZT4 in each renal cell type (colored dots). Dot size is proportional to the number of nuclei attributed to the cell type. (D) Volcano plot of differential mRNA expression between cKOt and Ctrl at ZT4, based on pseudo‐bulk analysis of the single‐nucleus (SN) dataset. mRNAs with significant fold changes (adjusted *p* < 0.05, threshold shown as dotted line) are highlighted in blue (less abundant in cKOt) or red (more abundant in cKOt). Numbers and percentages of differentially expressed mRNAs are indicated at the top of the plot. (E) UMAP generated from mRNAs in Ctrl and cKOt mice, ZT4. Nuclei are colored by sample (blue hues: Ctrl, red hues: CKOt). (F) Number of mRNAs more abundant (*y*‐axis) versus less abundant (*x*‐axis) in cKOt than in Ctrl mice at ZT4 in each renal cell type (colored dots). Dot size is proportional to the number of nuclei attributed to the cell type. (G) Volcano plot of differential lncRNA expression between cKOt and Ctrl at ZT16, based on pseudo‐bulk analysis of the single‐nucleus (SN) dataset. lncRNAs with significant fold changes (adjusted *p* < 0.05, threshold shown as dotted line) are highlighted in blue (less abundant in cKOt) or red (more abundant in cKOt). Numbers and percentages of differentially expressed lncRNAs are indicated at the top of the plot. (B) UMAP generated from lncRNAs in Ctrl and cKOt mice, ZT4. Nuclei are colored by sample (blue hues: Ctrl, red hues: CKOt). (H) UMAP generated from lncRNAs in Ctrl and cKOt mice, ZT16. Nuclei are colored by sample (blue hues: Ctrl, red hues: CKOt). (I) Number of lncRNAs more abundant (*y*‐axis) vs. less abundant (*x*‐axis) in cKOt than in Ctrl mice at ZT16 in each renal cell type (colored dots). Dot size is proportional to the number of nuclei attributed to the cell type. (J) Volcano plot of differential mRNA expression between cKOt and Ctrl at ZT16, based on pseudo‐bulk analysis of the single‐nucleus (SN) dataset. mRNAs with significant fold changes (adjusted *p* < 0.05, threshold shown as dotted line) are highlighted in blue (less abundant in cKOt) or red (more abundant in cKOt). Numbers and percentages of differentially expressed mRNAs are indicated at the top of the plot. (K) UMAP generated from mRNAs in Ctrl and cKOt mice, ZT16. Nuclei are colored by sample (blue hues: Ctrl, red hues: CKOt). (L) Number of mRNAs more abundant (*y*‐axis) versus less abundant (*x*‐axis) in cKOt than in Ctrl mice at ZT16 in each renal cell type (colored dots). Dot size is proportional to the number of nuclei attributed to the cell type.

In parallel, to investigate whether renal lncRNA expression depends on the intrinsic circadian clock, we compared cKOt versus Ctrl mice at ZT4 and at ZT16. In pseudo‐bulk analysis of SN data, we found that 63 lncRNAs (1.31% of lncRNAs) were differentially expressed (either upregulated or downregulated) between cKOt and Ctrl mice at ZT4 (Figure 3A). In a UMAP projection, the nuclei from both genotypes grouped together, though here again, less precisely for PCT nuclei than for other cell types (Figure 3B and Figure S3A for cell‐type identities in the UMAP). As expected, most lncRNAs deregulated in cKOt were found in tubular segments, primarily PCT and PST, whereas only a few changes were detected in vascular and mesangial/pericytes populations (Figure 3C; Table S7). For comparison, 319 mRNAs (1.95%) were differentially expressed at ZT4 in cKOt pseudo‐bulk data (Figure 3D). A UMAP of mRNAs displays robust overlap of nuclei from both genotypes for most cell types, with the exception of PCT and PST, where the nuclei from cKOt and Ctrl mice were visually separated into distinct strand‐like sub‐structures of cell clusters (Figure 3E). Analysis by cell type confirmed that most changes occurred in these two tubular segments, with a larger proportion of mRNAs downregulated rather than upregulated in cKOt (Figure 3F; Table S7). At ZT16, 40 lncRNAs (0.84%) were differentially expressed between genotypes in pseudo‐bulk analysis (Figure 3G). In the UMAP from ZT16, the nuclei from the two genotypes overlap well for all cell types, although PCT nuclei display incomplete alignment (Figures 3H and S3B for cell‐type identities in the UMAP). Differentially expressed lncRNAs were almost exclusively found in epithelial cells at ZT16, especially in PCT and PST cells, as observed at ZT4, but also in TAL cells, with no clear directional bias between upregulated and downregulated lncRNAs (Figure 3I; Table S7). Finally, 159 mRNAs (1.00%) were found deregulated in cKOt mice at ZT16 (Figure 3J). Cell‐type‐specific analyses confirmed that these transcriptional changes mainly occurred in tubular cells identified as PCT, PST and TAL cells (Figure 3K,L; Table S7). Figure panels 3B, 3E, 3H, and 3K show the same time‐point‐separated UMAPs as panels S2A–D, but colored by sample rather than by cell type.

We repeated the same differential expression analyses of lncRNAs and mRNAs between ZT4 and ZT16 or between cKOt and Ctrl using the DS dataset, which consisted of 16 RNA samples selected from our previously published renal circadian bulk RNA‐Seq experiment [26] that we had resequenced at higher depth (see Section 4) in order to enhance the detection of lowly expressed transcripts. RNAs with low expression were excluded from the analysis at the stage of *p*‐value adjustment. We required ≥ 5 read counts in > 50% of replicate samples of at least one of the experimental groups being contrasted. Even though the detailed lists of significantly differentially expressed genes differed between the SN and the DS datasets, the global expression patterns described above for the single‐nucleus transcriptome were confirmed in the RNA‐Seq transcriptome. Using four whole kidney samples per circadian time and genotype, we identified 222 lncRNAs (6.3%) and 1228 mRNAs (7.5%) that were differentially expressed between ZT4 and ZT16 (Figure S3A; Tables S8 and S9). Three core‐clock genes (*Cry1*, *Rorc*, and *Nr1d1*) ranked at the very top of differentially expressed genes. Three more (*Dbp*, *Nfil3*, and *Per2*) were among the top 25, indicating enrichment of clock genes in the top 25 differentially expressed genes (*p*‐value of Fisher's one‐tailed exact test = 5.167^−14^; Table S4). Moreover, we observed robust temporal asymmetry in differentially expressed lncRNAs, but not in mRNAs, with ~82% of lncRNAs upregulated at ZT4. We also found 180 lncRNAs (5.1%) and 1774 mRNAs (10.9%) that were differentially expressed between cKOt mice and Ctrl mice at ZT4 (Figure S3B), while at ZT16, 380 lncRNAs (10.8%) and 2556 mRNAs (15.7%) were differentially expressed between genotypes (Figure S3C). In cKOt, more lncRNAs were upregulated than downregulated at both available time points, whereas for mRNAs, this was not the case.

Altogether, these results indicate that a non‐negligible proportion of lncRNAs, just like mRNAs, are altered in their expression level during the circadian cycle and upon disruption of the renal tubular clock. Deregulated lncRNAs as well as mRNAs are restricted mainly to the epithelial compartment. Interestingly, unlike mRNAs, lncRNAs tend to be more abundant at ZT4 than at ZT16 in control mice.

### 2.3 Circadian Rhythmicity Patterns of Renal lncRNAs

**FIGURE 4:**
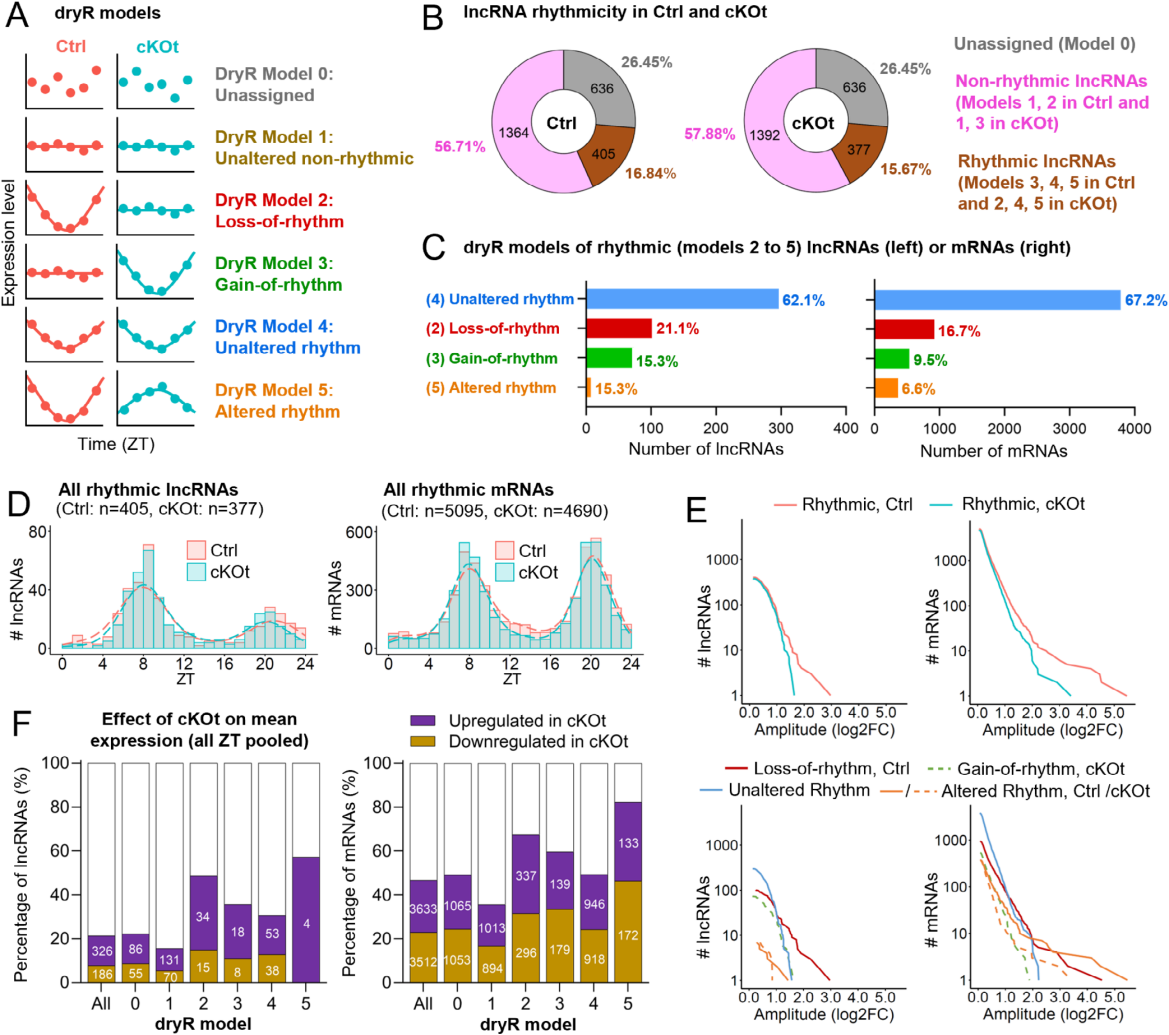
Circadian rhythmicity of renal lncRNAs and mRNAs in control and cKOt mice. (A) Circadian rhythmicity models across two genotypes. The dryR method assigns transcripts to one of its five models [1, 2, 3, 4, 5] using a Bayesian model selection strategy that considers expression data from both genotypes simultaneously to determine which RNAs are likely rhythmic in one, both, or neither genotype. For each transcript, sinusoidal curves and horizontal straight lines are fit to temporal expression data from both cKOt and Ctrl mouse genotypes; the model with the best fit across both (highest Bayesian Information criterion weight, BICW) is retained. Two models [4, 5] represent transcripts that are rhythmic in both genotypes: In Model 4, “unaltered rhythm”, both genotypes are fit by a sinusoidal with identical parameters; in Model 5, “altered rhythm”, acrophase, amplitude, or both differ between genotypes. Transcripts were left unassigned to any model if none of the five models had BICW > 0.65 (Model 0). (B) Numbers and percentages of rhythmic, nonrhythmic and unassigned lncRNAs in Ctrl and cKOt mice, based on dryR analysis, run with five replicate samples per genotype and six circadian time points (Zeitgeber Time, “ZT”). (C) Distribution of rhythmic lncRNAs (left) and mRNAs (right) among dryR Models 2 to 5. (D) Acrophase distributions of lncRNAs (left) and mRNAs (right) (rhythmic in Ctrl: Models 2, 4 and 5, red bars and red dashed line; rhythmic in cKOt: Models 3–5, blue bars and blue dashed line). Dashed lines are kernel density estimates. (E) Cumulative number of rhythmic lncRNAs (left) and mRNAs (right) as a function of oscillation amplitude. Top: Amplitudes of all transcripts rhythmic in Ctrl (Models 3–5) and in cKOt (Models 2, 4, 5). Bottom: Amplitudes per dryR model; dashed lines for cKOt (Models 3, 5); solid lines for Ctrl or both genotypes pooled (Models 2, 5; Model 4). Altered rhythm (model 5) has two separate lines for Ctrl and cKOt. (F) Number of renal lncRNAs (left) and mRNAs (right) significantly up or down in cKOt versus Ctrl mice. Differential expression analysis to compare genotypes was run using 30 samples per genotype, with a linear model that included a factor for genotype and a categorical control variable to adjust for circadian time points (R package *limma*). Significance threshold: Adjusted *p* < 0.05. Multiple testing correction method: Benjamini‐Hochberg. Column “All” (leftmost): Result from all transcripts. Columns 1–5: Result separated by circadian model from *dryR* analysis.

To evaluate the extent to which renal lncRNA expression is governed by the intrinsic circadian clock, we reanalyzed our previously published [26] circadian transcriptomic data generated from cKOt and Ctrl mice kidney samples harvested over a 24‐h period at six circadian time points spaced 4 h apart (ZT0, ZT4, ZT8, ZT12, ZT16, ZT20). The results have been implemented on our circadian kidney website to facilitate exploration of data (https://bix.unil.ch/circadian‐kidney/). Differential rhythmicity analysis was performed using the R package dryR (see Section 4) to classify transcripts into five groups according to their rhythmicity patterns in Ctrl and cKOt mice: [1] nonrhythmic; [2] rhythmic only in Ctrl; [3] rhythmic only in cKOt; [4] same rhythmicity pattern in both genotypes; [5] distinct rhythmicity patterns between the two genotypes. Transcripts were left unassigned if none of the five rhythmicity models were clearly dominant (Figure 4A). Transcripts belonging to models 2, 3, and 5 (with lost, gained, or altered circadian rhythmicity in cKOt versus Ctrl) are likely affected by disruption of the molecular core‐clock, whereas transcripts in model 4 retain their rhythmicity regardless of whether the core‐clock in tubules is intact.

This analysis revealed that 16.8% of lncRNAs, as well as 33.3% of mRNAs, are rhythmic in Ctrl kidney. In cKOt samples, 15.7% of lncRNAs and 30.6% of mRNAs are rhythmic (Figure 4B; Tables S10 and S11). Figure 4C shows the distribution of transcripts across rhythmicity models 2–5. About two‐thirds (62.1%) of rhythmic lncRNAs were classified under model 4 with unaltered circadian rhythm between genotypes (“system‐driven” lncRNAs). A fifth (21.1%) lost their rhythmicity (model 2, “BMAL1‐driven”), whereas 15.3% were nonrhythmic in Ctrl and acquired a circadian rhythm in cKOt mice (model 3). Only a handful of renal lncRNAs (1.5%) retained circadian oscillations in both genotypes while showing amplitude changes, phase shifts, or both after *Bmal1* deletion (model 5). The distribution of rhythmic mRNAs among dryR models paralleled that of lncRNAs: about two‐thirds of them (67.2%) were assigned to model 4, with decreasing numbers in models 2, 3, and 5 (16.7%, 9.5%, and 7%, respectively).

The distribution of acrophases along the circadian time axis is bimodal for lncRNAs and mRNAs. However, in lncRNAs, a higher proportion of rhythmic transcripts shows a peak of expression around ZT8 (inactive phase for mice) than around ZT20 (active phase for mice). This is not observed in mRNAs, whose acrophases are equally distributed between active and inactive phases (Figure 4D). Phase distribution is largely preserved in cKOt for both lncRNAs and mRNAs. Figure S4 shows acrophase distributions per rhythmicity model and enables a more detailed comparison of oscillatory phases between Ctrl and cKOt. In the mice's active phase, the circadian time when most lncRNAs reach their expression peak varies by dryR model. In Ctrl mice, lncRNAs in model 2 (with loss of rhythm in cKOt) peaked predominantly between ZT22 and ZT23. In cKOt mice, lncRNAs in model 3 (with acquired rhythm) peaked earlier, between ZT19 and 20. In model 4 (identical phase in both genotypes), the majority of lncRNAs peaked between ZT 20 and 21, corresponding to the histogram peak for mRNAs, which was stable across the three rhythmicity models 2 to 4.

As shown in Figure 4E, rhythmic lncRNAs display lower amplitudes than rhythmic mRNAs in Ctrl mice. Moreover, both rhythmic lncRNAs and mRNAs globally display lower amplitudes in cKOt than in Ctrl, which can be attributed to the loss of rhythmicity of RNAs of both types whose amplitudes were high in Ctrl (Model 2, red lines). Interestingly, lncRNAs that were rhythmic only in cKOt (Model 3, green line) display amplitudes comparable to those rhythmic in both genotypes (Model 4, blue line), whereas in mRNAs, Model 3 transcripts had lower amplitudes than Model 4 transcripts. Conversely, lncRNAs whose rhythmicity is retained but modulated in cKOt mice (Model 5, orange lines, solid and dashed) display similarly low amplitudes in both genotypes, while the amplitudes of mRNAs in Model 5 were different between the genotypes and higher than those of lncRNAs.

Since our previous work showed that *Bmal1* deletion affects not only the circadian rhythmicity of RNAs but also their mean expression levels across all circadian time points, we performed differential expression analysis of lncRNAs and of mRNAs in the Circ dataset, comparing all 30 cKOt versus 30 Ctrl samples. Our results indicate significant differential expression (adjusted *p* < 0.05) between the two genotypes in about 20% of lncRNAs and 45% of mRNAs. These percentages are even higher for transcripts that are rhythmic in one or both genotypes (models 2 to 5; 30%–50% of lncRNAs and 45%–80% of mRNAs), suggesting a link between rhythmicity and differential expression. About two‐thirds of affected lncRNAs were upregulated in cKOt mice, while mRNAs were downregulated as often as upregulated (Figure 4F).

## 3 Discussion

Our study described and compared the diversity and distribution as well as the circadian regulation of lncRNA and mRNA expression in the kidney of male mice at the single‐cell scale. Using snRNA‐seq, we detected a few hundred to a few thousand distinct annotated lncRNAs in each cell type we investigated. The lncRNAs found in each cell type consistently represented smaller proportions of the total number of lncRNAs than the corresponding proportions of mRNAs. Also, individual lncRNAs were, on average, restricted to fewer cell types than mRNAs, many of which are broadly expressed or ubiquitous across cell types. Nevertheless, the number of distinct lncRNAs per cell type is strongly correlated with the number of distinct mRNAs: some cell types are rich in both (including MD, PST, and bIC), and others are poorer (most notably, all immune cell types and the vasculature). We observed that the low expression of the majority of renal lncRNAs, which is consistent with findings in other organs [7], may, to a large extent, explain their higher cell‐type specificity compared to mRNAs. For both lncRNAs and mRNAs, cell‐type specificity and abundance are negatively correlated, but the proportion of lowly expressed (and consequently, the proportion of highly cell‐type specific) mRNAs is much smaller than it is for lncRNAs. A small subset of lncRNAs in our data was strictly limited to a single cell type (podocytes, MD, or beta‐intercalated cells), with no stray read counts detected in other cell types. All of them had only a handful of sequenced reads, summed across all cells from several samples, even in the cell type where they were present. In contrast, among the lncRNAs with higher expression levels that were concentrated in just one or in a few cell types, some reads were also found in other cell types. Determining whether these extra reads reflect functional activity or represent transcriptional or technical noise will continue to pose experimental challenges. Interestingly, a growing number of cell‐type–specific lncRNAs are reported to exert important functions despite low abundance, even in the cell types where they are enriched, and are being investigated as potential therapeutic targets [38, 39, 40]. For instance, Wisper and Fixer (described in the literature but currently not annotated in GENCODE or RefSeq) are expressed at low levels in cardiac fibroblasts and myofibroblasts but play key roles in cardiac fibrogenesis and tissue remodeling [41, 42]. Clustering analysis of lncRNA expression profiles sufficed to categorize renal cells into cell types, albeit less precisely than cell clustering based on mRNA expression. Aghagholzadeh et al. [42] have shown the same for the heart: lncRNAs alone can differentiate between cardiac cell types, although not quite as well as mRNAs alone, nor combined data comprising both. Considering the lower number of available features in lncRNAs and their generally much lower expression, which tends to produce less clear‐cut expression profiles, this level of cell‐type separability by lncRNAs is a relevant result that could not be counted upon a priori. Given that kidneys comprise dozens of highly differentiated cell types, adding selected lncRNAs to canonical marker gene lists used for cell‐type identification could prove valuable for single‐cell studies [31].

We characterized and compared the circadian rhythmicity of renal lncRNAs and mRNAs under physiological conditions and after disruption of the circadian clock in renal tubules. We observed a similar fraction of lncRNAs and mRNAs whose expression levels differed significantly between two circadian time points, one in the light phase (ZT4) and the other in the dark phase (ZT16). Our time‐resolved circadian RNA‐seq analysis revealed that 17% of lncRNAs and 33% of mRNAs are rhythmic in healthy murine kidney (Ctrl genotype), with mRNAs showing the highest amplitudes. This aligns with recent studies reporting rhythmicity in 5%–10% of detected lncRNAs in zebrafish larvae [43] or in mouse tissues [44, 45, 46]. In the kidney, rhythmic transcripts generally peak either in the middle of the inactive (light) phase (ZT4–ZT8) or in the middle of the active (dark) phase (ZT16–ZT20) [26]. Intriguingly, we observed that rhythmic lncRNAs peak preferentially during the inactive phase of the mice, unlike mRNAs. This light‐phase–skewed asymmetry seems to be mainly driven by a set of lncRNAs in the proximal segments of the tubule (PCT, PST) and in the TAL.

Only a handful of studies have explored the relationship between core‐clock components and the expression levels of rhythmic lncRNAs. Overall, these studies suggest that circadian oscillations in lncRNA expression may be partly driven by the core‐clock mechanism, as previously observed for mRNAs. Indeed, rhythmic lncRNAs possessing E‐box (enhancer box), D‐box (DBP box), or RORE response elements targeted by core‐clock transcription factors near their promoter regions have been experimentally validated or bioinformatically predicted in a few studies. Fan et al. [45] found that most loci of rhythmic lncRNAs in murine liver are bound by BMAL1 and REV‐ERBβ. Other studies demonstrated that specific rhythmic murine lncRNAs, such as *Platr4* in the liver and *AK028245* in the spleen, are upregulated when the core‐clock genes *N1rd1* (alias *Rev‐erb‐a*) and *Clock*, respectively, are knocked down [47, 48]. In the human kidney, ADIRF‐AS1 is rhythmic, and its promoter has been identified as a direct target of BMAL1 in U2OS cells [49]. In addition, some lncRNAs exhibit rhythms activated or modulated by light, whereas others have been shown to depend on the local clocks in peripheral organs [44, 45, 50]. In our study, 29% of lncRNAs and 22% of mRNAs that were rhythmic in the kidneys of Ctrl mice (Models 2, 4, 5) lost their rhythmicity in cKOt (Model 2), while 13% of lncRNAs and 13% of mRNAs that were nonrhythmic in Ctrl (Models 1, 3) became rhythmic in the absence of a functional tubular circadian clock (Model 3). The proportions of transcripts losing or acquiring circadian rhythms were thus similar in lncRNAs and mRNAs. Loss of rhythmicity in cKOt primarily affects transcripts with high amplitudes in Ctrl mice, indicating that high‐amplitude circadian oscillations of expression levels are more often clock‐driven rather than system‐driven in the kidney. On the other hand, transcripts that acquire rhythmicity in cKOt display amplitudes similar to or lower than those of transcripts whose circadian rhythms are not affected by genotype. As expected, the observed changes in rhythmicity are mostly limited to the tubular cell types in which the circadian clock is impaired. The distribution of acrophases of rhythmic lncRNAs along the circadian cycle is bimodal, with a larger number peaking in the light phase. Interestingly, this asymmetry toward the mice's inactive phase is observed in both Ctrl and cKOt, even for the lncRNAs that acquire rhythms following *Bmal1* inactivation (Model 3), indicating that it is a clock‐independent trend driven by systemic (cell‐extrinsic) time cues. In this vein, Park & Belden observed significant changes in histone 3 lysine 9 trimethylation (H3K9me3) levels between ZT4 and ZT16 at thousands of genomic loci in the zebrafish brain. Strikingly, these epigenetic DNA modifications associated with lowly transcribed heterochromatin were more abundant at ZT16 in young, healthy animals at both coding and noncoding loci. Gene expression generally showed the opposite pattern compared to H3K9me3 enrichment, as expected. This work suggests that the circadian rhythmicity of lncRNAs might be partly controlled through chromatin remodeling [13]. Finally, we found that the mean expression levels (averaged over 24 h) of many lncRNAs as well as mRNAs are significantly altered in cKOt mice compared to Ctrl, and this is even more frequent for rhythmic than for nonrhythmic transcripts. However, whereas renal mRNAs are equally likely to be upregulated or downregulated upon molecular clock disruption, lncRNAs are more often upregulated. This suggests that the cell‐intrinsic circadian clock not only modulates the rhythmicity but also decreases the expression of many lncRNAs.

Interestingly, some lncRNAs were found to be involved in circadian rhythm regulation [50]. Thus, among 424 rhythmic lncRNAs in the mouse kidney, Fan et al. demonstrated that *lnc‐Crot* is directly regulated by BMAL1 and REV‐ERBα and spans a super‐enhancer region, which in turn may help regulate the rhythmic expression of other genes. The lnc‐Crot locus forms chromatin loops to several other distant loci on the same chromosome, recruits clock transcription factors in physical proximity to these other sites, and raises the likelihood of rhythmic expression of genes near these sites. In the same study, hundreds of oscillatory lncRNAs were found to overlap with enhancer or super‐enhancer DNA motifs as well [45].

Among the lncRNAs that were rhythmic and/or regulated by the circadian clock in our datasets, only a handful have well‐established functions. Among them, *Gas5*, a known repressor of the glucocorticoid receptor [51], gains rhythmicity in cKOt mice, with its highest expression measured at ZT16. Considering the major effect of the glucocorticoid receptor's activation on peripheral clocks [52], the acquired Gas5 rhythmicity upon clock disruption might contribute to perturbations in the rhythmicity of the renal transcriptome in cKOt mice. *Gas5* has also been shown to interfere with renal apoptosis and fibrosis by modulating multiple miRNA pathways [53, 54, 55, 56], suggesting that *Gas5* modulation in cKOt mice might affect their ability to cope with kidney injury. Of note, the lncRNA *Neat1*, which is involved in numerous pathological conditions and contributes to acute kidney injury [57, 58], also gains rhythmicity in cKOt mice, with a similar peak time as *Gas5*. Finally, among lncRNAs with significantly different mean expression levels between cKOt and Ctrl (averaged over 24 h), *HK1os* is 30% more abundant in cKOt. This observation is of particular interest because HK1os is transcribed on the opposite strand of the HK1 gene that encodes the glycolytic hexokinase enzyme. Thus, *HK1os* upregulation might play a role in the reduced renal glycolysis (accompanied by increased renal gluconeogenesis) observed upon tubular clock disruption that we have previously described [30, 59]. However, it remains essential to recognize that the mechanistic roles of lncRNAs often vary between cell types and depend on their expression levels and subcellular localization [5, 60]. Functional descriptions must be contextualized, and a priori generalizations to other cell types, tissues or organs should be avoided. In line with these considerations, establishing causal roles for the lncRNAs highlighted in this study will require functional perturbation, for instance using antisense oligonucleotides or CRISPR interference approaches [61].

While our findings provide new insights into the expression landscape of lncRNAs in the kidney and raise new questions for future investigation, they should be interpreted with respect to certain limitations inherent to our experimental design. First, our single‐nucleus transcriptomic analysis was performed on isolated nuclei. Single‐nucleus RNA‐seq is a suitable approach for lncRNA profiling, as many lncRNAs are enriched in the nucleus [7, 62, 63]. In the kidney, this method helps minimize dissociation‐induced biases known to affect conventional sRNA‐seq [64]. However, studies have highlighted that the cytoplasm harbors a large population of lncRNAs as well [63, 65], including preferentially cytoplasmic ones, which are not captured in our nuclear data. Second, the evolutionary conservation of lncRNAs is limited, and many are known to be species‐specific [12]. As such, caution should be exercised when extrapolating our findings from mouse to human, particularly with regard to the functional relevance of specific lncRNAs in renal circadian regulation. Third, only male mice were included in our experiments, and our findings may not be generalizable to females. Indeed, sex‐specific differences in BMAL1 transcriptional effects [66] and in lncRNA expression [67] have been reported in other contexts, and there is mounting evidence of sex‐specific differences in circadian physiology [68]. In the latter study, a large‐scale analysis of circadian transcriptomic data from more than 40 human tissues revealed that the number of rhythmic genes is approximately twofold higher in women than in men. Furthermore, distal tubule deletion of Bmal1 in the mouse kidney has differential impact on males and females [69]. Altogether, these data suggest that the repertoire of rhythmic lncRNAs, as well as their abundance, might differ in female compared to male mouse kidneys.

Fourth, describing and quantifying lncRNA expression trends globally for a tissue is aiming at a moving target. We have shown that RNA‐seq enables the detection of reads from up to 7553 of the 11 608 sequences categorized as lncRNAs in the Ensembl annotation of the mouse genome that we used (Grcm 39, Release 110). Given that Ensembl currently contains only a fraction of all lncRNAs (by comparison, the NONCODEV6 database, which merges data from several databases and annotation sources including Ensembl, contained 131 974 mouse lncRNA transcripts at its last update in 2021 [70]), these numbers suggest that tens of thousands of lncRNAs might be expressed in the kidney. Limiting ourselves to RNAs found in a standard annotation has the advantages of (a) expert curation of the loci to which our reads were mapped (the genome assembly and experimental sequence evidence must be available, filtering criteria must be met), and (b) better reproducibility of our findings (www.ensembl.org). However, this decision naturally excluded a much larger number of lncRNAs from our count statistics than would relying on a de novo assembly or on sequence reference data from less strictly curated sources such as Ensembl. Fifth, an open question, which, to our knowledge, has not been widely discussed, is whether the lower prevalence and higher specificity of diurnal rhythms in lncRNAs compared to mRNAs is to some extent an artifact due to low expression, similar to the elevated cell‐type specificity of lncRNAs. The low number of read counts in many lncRNAs could inflate false negatives, as curve fitting will carry more uncertainty. Finally, although BMAL1 is a core component of the circadian clock, it also acts as a transcription factor and translation factor with clock‐independent functions [71]. Moreover, the circadian clock relies on several regulatory loops, and even in the absence of one component, such as BMAL1 in our work, some RNAs whose rhythmicity depends on the cell‐intrinsic clocks in renal tubules might remain oscillatory [72]. Therefore, the transcriptional effects observed in our *Bmal1* knockout model may not exclusively and fully reflect changes induced by circadian clock disruption, and comparisons with additional genetic or environmental models of clock perturbation would be valuable.

## 4 Methods

Additional details are presented in Supplemental Methods.

### 4.1 Sex as a Biological Variable

Male mice were examined because we relied on RNA samples and sequencing data from our previous study performed solely on males [26]. We address this limitation in Section 3.

### 4.2 Animal Experiments

Experiments were performed on male Bmal1^lox/lox^/Pax8‐rtTA/LC‐1 mice (referred to as cKOt) and Bmal1^lox/lox^ (referred to as Ctrl) as previously described [26]. After conditional inactivation of *Bmal1* (*Arntl*) in the cKOt genotype, mice were kept in circadian boxes under 12‐h light/12‐h dark cycles for 2 weeks before sacrifice at 14 weeks of age. Experimental timeline, euthanasia and kidney sample preparation are documented in Supplemental Methods.

### 4.3 Renal Nuclei Isolation and snRNA‐Seq

Renal nuclei were isolated, DAPI‐labeled and sorted (see Supplemental Methods), then encapsulated using Chromium Next GEM Single Cell 3′ Reagent Kits v3.1 (Dual Index) (10X Genomics). GEM generation, cDNA amplification and library preparation were performed according to the manufacturer's protocol. Sequencing was performed on an Illumina NovaSeq 6000 v1.5 flow cell with recommended settings. Around 10 000 nuclei were recovered per sample (Table S12). A total of 12 240 million reads were generated to reach a sequencing depth of ~150 000 reads per cell in each sample. Data were demultiplexed using bcl2fastq2 (v2.20, Illumina) and primary data analysis performed with Cell Ranger (v7.1.0, genome reference: refdata‐gex‐mm10‐2020‐A, 10X Genomics).

### 4.4 RNA‐Seq of Whole Kidney Samples

Existing RNA libraries were resequenced on an Illumina NovaSeq 6000 to a depth of 320 to 573 million reads per sample (Table S12). Reads were aligned to Grcm 39, Ensembl Release 110. Technical details are given in Supplemental Methods.

### 4.5 Experimental Design

We produced three renal transcriptomics datasets from cKOt and Ctrl mice. **SN**. Single nuclei were obtained from eight animals: two replicates per genotype from two circadian time points, ZT4 and ZT16 (4 h after light/resting phase onset, and 4 h after dark/active phase onset). Circ—Bulk RNA‐Seq, reused from prior work, comprised samples from 60 animals: five replicates per genotype from six circadian time points. DS—A subset of 16 RNA libraries, with four replicates per genotype from the two time points ZT4 and ZT16, was resequenced more deeply (see above) and used as an additional dataset.

### 4.6 Data Analysis

Data analyses were run in R (v4.3.1 and v4.3.2). Single‐nucleus data were processed using Seurat (v5.1.0), with cell‐type assignment executed manually. Table S13 and Figures S5 and S6 show the marker genes used. Comprehensive analysis details, including data thresholds, domain‐specific definition of the Gini coefficient and detailed Seurat workflows, are provided in Supplemental Methods. *Statistics*—Differential expression analyses were conducted with the R Bioconductor packages *DESeq2* (v1.40.2, DS dataset and v1.42.1, SN dataset) and *limma* (v3.56.2, Circ dataset). All statistical models included two genotypes and either two or six time points, depending on the dataset. Contrasts of interest comparing genotypes or time points were extracted from the models. *p*‐values were adjusted for multiple testing by the Benjamini‐Hochberg method. Adjusted *p* < 0.05 was considered statistically significant. Circadian analyses were performed with the R package dryR (v1.0.0), which classified transcripts into one of five circadian models using a Bayesian approach. Transcripts were assigned to a circadian model if they passed the threshold BICW > 0.65. Further details regarding both analyses are available in Supplemental Methods.

### 4.7 Study Approval

Animal experiments were approved by the Swiss cantonal (Vaud) and federal veterinary authorities (authorization #3488d to DF).

## Author Contributions

**Fanny Durussel:** investigation. **Yohan Bignon:** conceptualization, investigation, data curation, formal analysis, writing – original draft, writing – review and editing. **Leonore Wigger:** conceptualization, investigation, data curation, formal analysis, writing – original draft, writing – review and editing. **Muriel Auberson:** investigation. **Dmitri Firsov:** conceptualization, funding acquisition, writing – review and editing.

## Funding

This work was supported by the Swiss National Science Foundation, 320030‐231189.

## Conflicts of Interest

The authors declare no conflicts of interest.

## Supporting information


**Table S1:** Number of nuclei per cell type.

**Table S2:** Gini coef lncRNA vs. mRNA *t*‐test.

**Table S3:** SN pseudobulk DESeq2 mRNA.

**Table S4:** Clock gene enrichment in DESeq2 by Fisher tests.

**Table S5:** SN pseudobulk DESeq2 lncRNA.

**Table S6:** Number of DE lncRNA and mRNA per cell type ZT16vsZT4.

**Table S7:** Number of DE lncRNA and mRNA per cell type cKOt vs. Ctrl.

**Table S8:** DS DESeq2 lncRNA.

**Table S9:** DS DESeq2 mRNA.

**Table S10:** Circadian data stat results lncRNA.

**Table S11:** Circadian data stat results mRNA.

**Table S12:** Sequencing depth SN and DS.

**Table S13:** Marker genes for cell type annotation.

**Data S1:** Supporting data values: All data underlying figures provided in the manuscript.

**Data S2:** Supplemental methods.
**Figure S1:**: Diversity and specificity of lncRNAs in renal cells.
**Figure S2:**: Cell types in UMAPsof lncRNAs and mRNAs, split by ZT.
**Figure S3:**: Effects of circadian time and the circadian clock on renal lncRNA and mRNA expression in deeply sequenced bulk RNA‐seq data.
**Figure S4:**: Acrophase distribution of renal lncRNAs and mRNAs per dryR rhythmicity model.
**Figure S5:**: Renal marker genes delineating cell types of the nonimmune compartment.
**Figure S6:**: Renal marker genes delineating cell‐type groups of the immune compartment.

## Data Availability

Data underlying figures are provided in supporting data values (Data S1). RNA‐Seq data are available on the Gene Expression Omnibus (https://www.ncbi.nlm.nih.gov/geo/). Accession numbers: GSE331333 (SuperSeries for three datasets); SubSeries: GSE331323 (DS data), GSE331332 (SN data), and GSE331330 (Circ data). Selected results and circadian expression profiles of lncRNAs and mRNAs can be perused on the project website (https://bix.unil.ch/circadian‐kidney/). Feature plots of 273 known marker genes that were screened for cell type annotation, as well as Tables [Link], [Link], [Link], [Link], [Link], [Link], [Link], [Link], [Link], [Link], [Link], [Link], were deposited on Zenodo under DOI: https://doi.org/10.5281/zenodo.20358692.
