# Supplementary material for: Expression Landscape and Circadian Regulation of lncRNAs in the Kidney
Source: Acta Physiol (Oxf). 2026 Jul 3;242(8):e70273. doi: 10.1111/apha.70273 (PMC13332011; doi:10.1111/apha.70273)
Supplement: Supplementary file 15 — Data S2: Supplemental methods. Figure S1:: Diversity and specificity of lncRNAs in renal cells. Figure S2:: Cell types in UMAPsof lncRNAs and mRNAs, split by ZT. Figure S3:: Effects of circadian time and the circadian clock on renal lncRNA and mRNA expression in deeply sequenced bulk RNA‐seq data. Figure S4:: Acrophase distribution of renal lncRNAs and mRNAs per dryR rhythmicity model. Figure S5:: Renal marker genes delineating cell types of the nonimmune compartment. Figure S6:: Renal marker genes delineating cell‐type groups of the immune compartment. [file APHA-242-e70273-s014.pdf]

Expression landscape and circadian regulation of lncRNAs in the kidney

Leonore Wigger, Fanny Durussel, Dmitri Firsov and Yohan Bignon

SUPPLEMENTAL METHODS

Animal experiments. Experiments were performed on Bmal1$^{lox/lox}$/Pax8-rtTA/LC-1 mice (referred to as cKOt mice) and Bmal1$^{lox/lox}$ (referred to as Ctrl mice) bred on the C57BL/6J background. Mice were maintained on a 12-hour light/12-hour dark cycle with *ad libitum* access to drinking water and a standard chow diet (KLIBA NAFAG diet 3800). To induce Cre recombinase expression and inactivation of the *Bmal1* (*Arntl*) gene in renal tubules, eight-week-old cKOt mice and their Ctrl littermates were exposed to 2 mg/ml of Doxycycline and 20 mg/ml sucrose in their drinking water for 14 days. At 10 weeks of age, the mice were taken off Doxycycline and maintained without treatment for two weeks. At 12 weeks, they were transferred to circadian boxes with individually programmable light cycles. Light on/off times were adjusted relative to the planned ZT of kidney harvesting to enable sacrifice at 11 am. For example, for mice scheduled for sacrifice at ZT 4, the light phase began at 7 am. All mice underwent two weeks of circadian entrainment in the boxes. At 14 weeks of age, the mice were deeply anesthetized by intraperitoneal injection of 10 mg/kg xylazine (Rompun, Bayer) and 100 mg/kg ketamine (Ketanarkon, Streuli) and euthanized by intracardiac perfusion. Blood was flushed from the tissues by intracardiac injection of PBS supplemented with 30 mM KCl and 0.01% heparin (Sigma-Aldrich, ref. H3149). Both kidneys were harvested, sliced, flash-frozen in liquid nitrogen and stored at -80 °C. For the bulk RNA-seq experiment, the kidneys were bisected transversely, and one half of the left kidney was used for RNA extraction. For the single-nucleus experiment, the kidneys were decapsulated and cut transversely to yield central cross-sections of about 25 mg each, containing cortex, outer medulla and inner medulla. One section was used to verify *Bmal1* inactivation by PCR and electrophoresis, while nuclei were isolated from the other section.

Renal nuclei isolation. Frozen renal slices were immersed in 2 mL of ice-cold lysis buffer (Sigma-Aldrich, ref. NUC-101) supplemented with RNase inhibitors (RNasin, Promega, ref. N2615 at 100 U/mL, and RiboLock RNase Inhibitor, Thermo Fisher, ref. EO0384 at 50 U/mL) and a cocktail of protease inhibitors (Mini cOmplete ULTRA Tablets, Roche, ref. 5892791001; 1 tablet per 8 mL of buffer). Renal tissue was then homogenized at −20 °C for 2.5 minutes using a TissueLyser (Qiagen) and stainless-steel beads (Qiagen, ref. 69989, 5 mm) set at high speed (25 Hz; 1,500 oscillations per second) to disrupt cell plasma membranes. An additional 2 mL of ice-cold lysis buffer was added to the lysate. To separate intact cells and debris from isolated nuclei, the lysate was filtered through a 40 µm cell strainer (Sigma, Corning cell strainer, ref. CLS431750) and centrifuged at 4 °C for 5 minutes at

500 × g. The pellet was resuspended in 2 mL of ice-cold lysis buffer (Sigma-Aldrich, ref. NUC-101), incubated on ice for 5 minutes, and centrifuged at 4 °C for 5 minutes at 500 × g. It was then gently resuspended in 1.2 mL of ice-cold resuspension buffer (phosphate-buffered saline supplemented with 2% bovine serum albumin, RNasin, and RiboLock RNase inhibitors) and purified by gravity filtration through four 20 µm cell strainers (pluriSelect, pluriStrainer Mini 20 µm, ref. 43-10020-40; one strainer per 300 µL of nuclei suspension). DAPI was added at a final concentration of 1 µg/mL, and nuclei were counted using a fluorescence microscope. To limit contamination with ambient RNAs derived from disrupted nuclei or from cells' cytoplasms, and to eliminate nuclei with compromised membranes, doublets or clumps, we sorted 50.000 DAPI-labelled nuclei from the nuclei solution. Nuclei were sorted on a MoFlo Astrios EQ cell sorter (Beckman Coulter) using a 50 µm nozzle tip at a sheath pressure of 70 psi. Sorted nuclei were collected in ice-cold resuspension buffer. The number of nuclei passing the cell sorter was kept low (30–60 events per second) to reduce contamination with ambient RNA further. To assess nuclei concentration, nuclei suspensions were diluted 40-fold, stained with propidium iodide (PI) and counted using a Moxi V cell counter (Orflow).

RNA Sequencing. RNA libraries for the SN and DS datasets were sequenced on a NovaSeq 6000 v1.5 (Illumina) following the manufacturer's recommendations. Paired-end reads were obtained.

Bulk RNA-seq data processing. The FASTQ files from the **Circ** dataset, which we previously published (1), were reprocessed for this work, and the **DS** data, generated by deeper sequencing of a subset of the same RNA libraries, were processed using the same pipeline. Initial preprocessing and quality control were performed with the command-line tool fastp (v0.23.2). Several filtering options were used to trim reads and to discard low-quality reads:

```
fastp \
--in1 $R1 --out1 $R1CLEAN \
--in2 $R2 --out2 $R2CLEAN \
--length_required 35 \
--adapter_sequence=AGATCGGAAGAGCACACGTCTGAACTCCAGTCA \
--adapter_sequence_r2=AGATCGGAAGAGCGTCGTGTAGGGAAAGAGTGT \
--cut_mean_quality 15 \
--cut_right \
--correction \
--trim_poly_g \
--trim_poly_x \
--n_base_limit 5 \
```

The minimum read length required was set to 35 (--length_required 35); adapters were trimmed by the default overlap analysis, with Illumina adapter sequences supplied for cases where this fails; trimming of low-quality bases was performed with a sliding window approach, requiring a mean quality phred score of 15 across a sliding window of default size 4 and dropping the bases in the window and to its right otherwise (--cut_mean_quality 15, --cut_right); bae correction in overlapped regions of paired ends was enabled; polyG and polyX tails were trimmed (--trim_poly_g, --trim_poly_x); and reads with >5 "N" base calls were removed (--n_base_limit 5).

Alignment to the Ensembl genome *Mus_musculus.GRCm39.110* was performed with the command-line tool RSEM (v1.3.3), using the STAR method (--star). Gene count matrices from RSEM were used for downstream analyses.

Cell-type assignment in single-nucleus data. Single-nucleus data was processed using the R package Seurat (v5.1.0) in R (v4.3.2). The SN dataset comprised eight samples from eight mouse individuals, with two replicate mice per experimental condition (Ctrl-ZT4, Ctrl-ZT16, cKOt-ZT4, cKOt-ZT4). Initial filtering required that each feature be expressed in at least three cells, that each cell had at least 500 expressed features (nFeature_RNA) and 800 feature counts (nCount_RNA), and no more than 2% mitochondrial RNA. Mitochondrial genes were removed. Seurat normalization (division by the sum of reads per cell) was applied with default settings. All principal component analyses (PCA) were run on the 2'000 most variable features selected by the vst method.

Cell types were identified manually in a multi-step approach, relying on both computational analyses and biological expertise.

1. We generated UMAPs from all eight samples, using only protein-coding genes, testing a range of numbers of principal components from PCA (20-1000). We proceeded with the UMAP generated from 1000 principal components, chosen to retain roughly 80% of the variance and to optimize visual separation of cell types, especially for rare populations.
2. We decided not to use Seurat's built-in data integration step for combining multiple samples. The data points from all samples were well superimposed across most regions of the UMAPs, and we interpreted the limited areas showing separation between samples as reflecting actual, biologically relevant differences in gene expression.
3. We generated 347 individual feature plots of previously known marker genes, visualizing their expression levels (available on Zenodo, DOI: 10.5281/zenodo.20358692). We included all 265 marker genes listed in (2), a resource and review paper on mouse kidney cell types, and added eight additional well-known markers from studies of mouse and human kidneys (Atp16b, Crip1, Slc34a3, Aqp7, Slc5a1, Adgrf1, Rhcg).. We included all marker genes listed in (2), a review paper on mouse kidney cell types, and added several additional well-known markers from studies of mouse and human kidneys.
4. We looked through all feature plots to manually draw approximate boundaries between cell types with known markers into the UMAP (with pen on paper printouts). Supplemental File S13 provides a curated set of 131 markers that were specific to or strongly over-expressed in distinct UMAP regions. Certain cell types were not sufficiently differentiated in the UMAPs to allow separate annotations (for example, mesangium and pericytes could not be distinguished and were lumped into a single cell category, as were all cell types of the vasculature). In contrast, other cell types could be subdivided more finely, in particular those of the distal renal

tubule (for example, the cortical and medullary parts of the collecting duct, MCD and CCD, were distinguishable and labeled as two different cell types). Because the epithelial cell types that make up the renal tubule form a continuum, drawing boundaries between them is somewhat arbitrary. This reflects the biological reality of intermediate cell types found between different tubule segments. We settled on 21 cell types, some of which were categories of a few very similar cell types, and manually labeled the point clouds delineated in the UMAP with cell-type names.

5. We performed unsupervised clustering analysis in Seurat with a range of resolutions. We retained the clustering resolution 2.2, which fit well with the manually drawn cell-type boundaries, and labeled the clusters with cell-type names. For some cell types, we merged multiple clusters. Seven clusters could not be assigned to any cell type and were labeled N/A. In addition, some clusters included small "satellite" point clouds in the UMAP, where we had doubts about whether they belonged to the same cell type. We excluded three such satellites from their respective clusters using Seurat's cell selector tool: two small satellites of the SMC/JCG cluster were excluded because the key marker gene *Ren1*, which encodes the highly specific protein Renin, was not expressed in them, and one small satellite of the myeloid cluster was excluded as it jumped between two different immune cell clusters at different clustering resolutions (myeloid immune cells and TC/NK) and shared markers with both.
6. We performed differential expression analysis with the function FindAllMarkers, comparing gene expression in each cluster against that in all other clusters combined. We checked the top differentially expressed genes with the highest fold change and smallest p-values. This analysis allowed us to identify one of the unlabelled clusters as urothelium, based on strong overexpression of Upk1b, a well-known supported by strong overexpression Upk1b, a known marker gene that that had not been in our initial marker list.
7. After these manual modifications, the cell type assignment obtained from mRNA expression across all eight samples was considered definitive and applied in all subsequent analyses performed with mRNAs or with lncRNAs. Dot plots representing relative expression levels and percentage of expressing cells per cell type are available in Figures S5 and S6.

For UMAP generation from lncRNAs alone, 640 principal components were used, capturing about 83% of the data variance when all eight samples were used, and 78% of the variance when only the four Ctrl samples were included. For mRNAs, the variance that was captured by the first 1000 principal components remained at 80% regardless of whether PCA was performed on all eight samples or on the four Ctrl samples only.

The Gini coefficient as a measure of cell-type specificity. The Gini coefficient is a well-established tool in economics for quantifying income (or wealth) inequality among a nation's or geographic region's inhabitants. It was initially proposed by the statistician Corrado Gini in 1912 (3). A Gini coefficient of 0 signifies perfect equality, where all individuals have the same income. A Gini coefficient of 1 means that a single individual has all the income and the others have none. The Gini coefficient represents the degree to which an income distribution deviates from an equal distribution. It has a geometric definition based on the Lorenz curve, a curve derived from empirical data that plots cumulative income against population percentiles. It is defined as twice the area between the Lorenz curve and the diagonal line that represents perfect equality. Here, a variant of it is used to quantify the cell-type specificity of genes. Instead of assessing income distribution among people (which allows comparisons between nations), we assess read count distribution among cell types (which allows comparisons between genes). In place of income received by an individual, we define the basic value as the proportion of cells within a cell type that express a gene with ≥1 read count. In our case, a Gini coefficient of 1 signifies maximal cell-type specificity: a gene's expression is entirely restricted to a single cell type. A Gini coefficient of 0 indicates that all cell types have equal proportions of cells expressing the gene. The following examples illustrate Gini coefficient thresholds:

- If **80%** of income is with **20%** of a population, the Gini coefficient for this population is at least (0.8-0.2) = **0.60**
- If **80%** of cells expressing a gene come from 1 cell type out of 20 **(5% of cell types)**, the Gini coefficient for this gene is at least (0.8-0.05) = **0.75**
- If **95%** of cells expressing a gene come from 1 cell type out of 21 **(5.0476% of cell types)**, the Gini coefficient for this gene is at least (0.95-5.0476) = **0.902**

The Gini coefficient in its original form (2x the area between the Lorenz curve and the equality line) is downward-biased for small sample sizes. A simple adjustment has been proposed (4): multiplying the Gini coefficient by *n/(n-1)*. This brings the Gini index to a measure ranging from exactly 0 to exactly 1. It diminishes the first-order bias: Typically, less than 5% bias remains for sample sizes between 5 and 10. In our study, we used the Gini() function implemented in the R package DescTools (0.99.58), which applies this adjustment by default. Our sample size is *n*=21 (number of cell types), and the Gini coefficients were thus multiplied by 21/(21-1) = 1.05 to unbias them.

Differential Expression Analysis. For the SN dataset, pseudo-bulk read count tables with one column per sample and one row per lncRNA or mRNA were generated using the standard procedure, summing the read counts for each gene over all cells in that sample. SN pseudo-bulk read counts and DS bulk read counts were analyzed in the same way with the R Bioconductor package *DESeq2* (v1.40.2, DS

dataset and v1.42.1, SN dataset). The DESeq function, which performs data normalization and a Wald significance test assuming a negative binomial distribution, was applied to the full, unfiltered read count matrices, using default parameters. No beta prior was used (betaPrior=FALSE). In each data set, two separate, identical analyses were then run on lncRNAs and mRNAs. Four contrasts were extracted from the statistical model for pairwise comparisons of experimental groups:

```
cKOt_ZT04 vs. Ctrl_ZT04 (the genotype effect at ZT04)
cKOt_ZT16 vs. Ctrl_ZT16 (the genotype effect at ZT16)
Ctrl_ZT16 vs. Ctrl_ZT04 (the time effect for Ctrl)
cKOt_ZT16 vs. Ctrl_ZT04 (the time effect for cKOt)
```

Fold changes and p-values were obtained for all features. P-values were adjusted for multiple testing by the Benjamini-Hochberg method. Prior to p-value adjustment, rows were filtered using custom-defined criteria to exclude lowly expressed features. The filtering was performed separately for each contrast, so the total number of features with adjusted p-values differed between contrasts. Only these were considered to have a valid result and were counted in the reporting of numbers and percentages of differentially expressed lncRNAs or mRNAs. The filtering criteria for this step were defined as follows: In the SN dataset, which had two replicates per experimental condition, features were included if at least one of the two groups being compared had ≥5 pseudo-bulk read counts in both replicates. In the DS dataset, which had four replicates per experimental condition, features were included if at least one of the two groups being compared had ≥5 read counts in ≥3 replicates. Volcano plots were generated using GraphPad Prism v10.

In the Circ dataset, differential expression was calculated between cKOt and Ctrl using the R Bioconductor package *limma* (v3.56.2). Prior to statistical analyses, TMM normalization was applied, and batch effects were corrected using the RUVs method, implemented in the R Bioconductor package *RUVSeq* (v1.34.0), as described in (1). The data comprised samples from six ZT for each of the two genotypes, with five replicates per experimental group, for a total of 60 samples. Lowly expressed features were filtered out prior to differential expression analysis by the following criterion: At least one experimental group had to have ≥5 read counts in ≥3 replicates for inclusion. A moderated t-test from *limma* (based on a linear model) was performed to compare expression levels averaged over all time points between the two genotypes. To obtain the genotype effect independent of the circadian time effect, we factored out the circadian time effect by including a categorical control variable for the circadian time points in the linear model. P-values were adjusted for multiple testing by the Benjamini-Hochberg method.

REFERENCES

1. Bignon Y et al. Multiomics reveals multilevel control of renal and systemic metabolism by the renal tubular circadian clock. *J Clin Invest*. 2023;133(8):e167133. doi:10.1172/JCI167133
2. Balzer MS et al. How Many Cell Types Are in the Kidney and What Do They Do? *Annu Rev Physiol*. 2022;84:507-531. doi:10.1146/annurev-physiol-052521-121841

3. Gini, C. Variabilita e mutabilita: Contributo allo studio delle distribuzioni e relazioni statistiche. Tipogr. di P. Cuppini. 1912
4. George Deltas. The Small-Sample Bias of the Gini Coefficient: Results and Implications for Empirical Research. *Rev. Econ. Stat.* 2003; 85(1): 226-234. doi:10.1162/rest.2003.85.1.226

SUPPLEMENTAL FIGURES

Figure S1: Diversity and specificity of lncRNAs in renal cells.
Figure S2: Cell types in UMAPsof lncRNAs and mRNAs, split by ZT.
Figure S3: Effects of circadian time and the circadian clock on renal lncRNA and mRNA expression in deeply sequenced bulk RNA-seq data.
Figure S4**:** Acrophase distribution of renal lncRNAs and mRNAs per dryR rhythmicity model.
Figure S5: Renal marker genes delineating cell types of the non-immune compartment.
Figure S6: Renal marker genes delineating cell-type groups of the immune compartment.

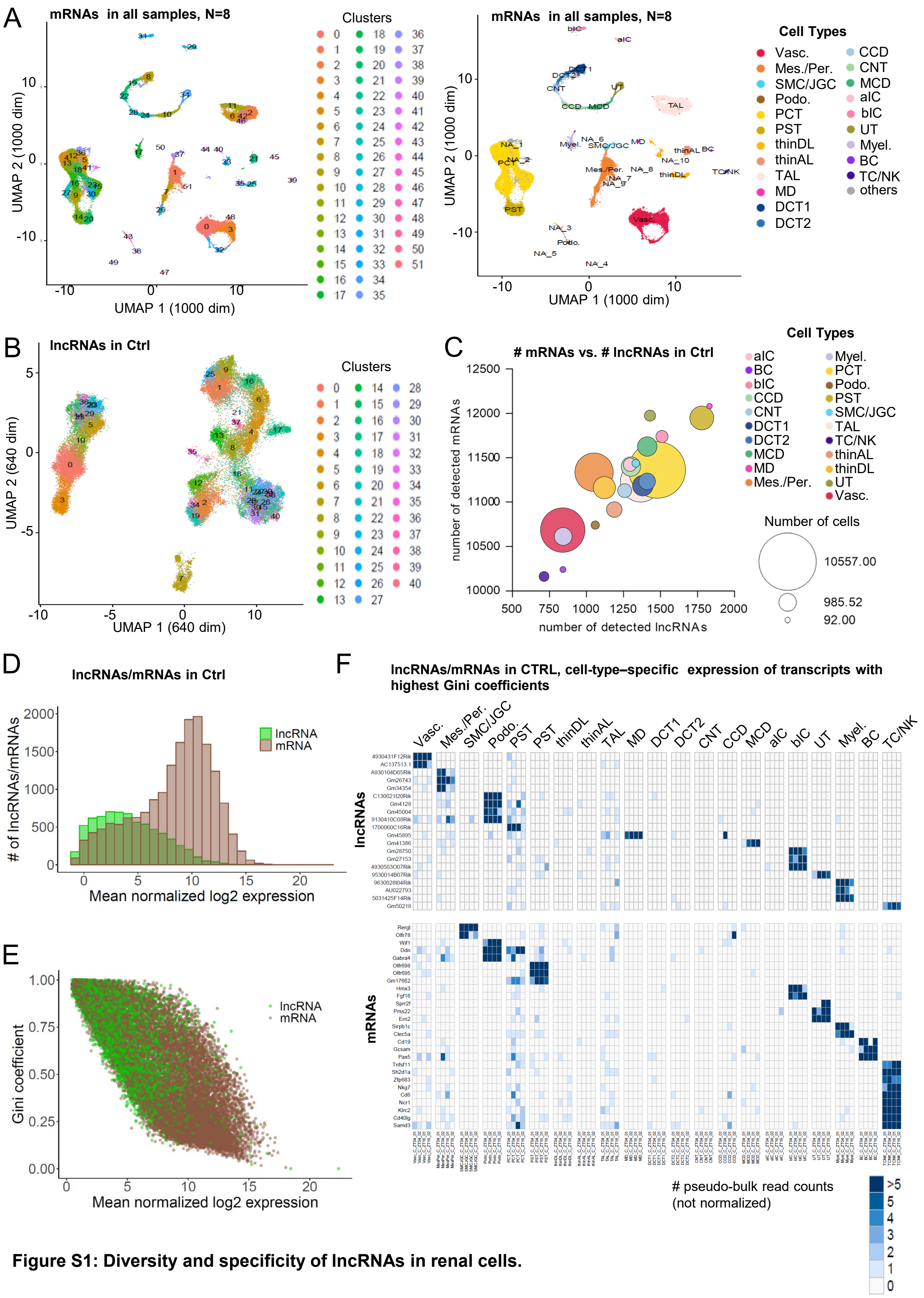

Figure S1: Diversity and specificity of lncRNAs in renal cells.

Figure S1: Diversity and specificity of lncRNAs in renal cells. A. UMAP generated from renal mRNAs in Ctrl and cKOt mice, ZT4 and ZT16 pooled (*N*=8). Nuclei are colored by cluster labels from unsupervised clustering analysis performed exclusively on mRNAs (left) or by their manually attributed cell type/category based on this clustering (right). **B.** UMAP generated from renal lncRNAs, using Ctrl mice only, ZT4 and ZT16 pooled (*N*=4). Nuclei are colored by the same cell type labels as in panel A (right), obtained from analysis of mRNAs. **C.** Number of mRNAs (y-axis) vs. lncRNAs (x-axis) in Ctrl mice for each renal cell type (colored dots). Dot size is proportional to the number of nuclei attributed to the cell type. **D.** Superposed histograms of expression levels of lncRNAs (green) and mRNAs (brown) in Ctrl mice. Transcripts are grouped into bins based on their mean expression across samples. Data processing: Pseudo-bulk from SN data, normalized with Deseq2 and transformed with regularized log2 (rlog). No filtering for low expression was applied. **E.** Relation between Gini coefficient and expression level of mRNAs (blue dots) and lncRNAs (red dots) in Ctrl mice. Data processing: Pseudo-bulk from SN data, normalized with Deseq2 and transformed with regularized log2 (rlog). Filtering for low expression: at least 1 count in one of 21 cell types. **F.** Heatmaps showing expression per cell type for the most cell-type–specific lncRNAs (top) and mRNAs (bottom), having a Gini coefficient ≥ 0.95. All are lowly expressed, with read counts per cell type in the single digits. Data processing: pseudo-bulk from SN data, read counts without normalization. Filtering for low expression: At least 5 counts in two replicates in at least one time point in control mice. See Figure 1A for cell type abbreviations.

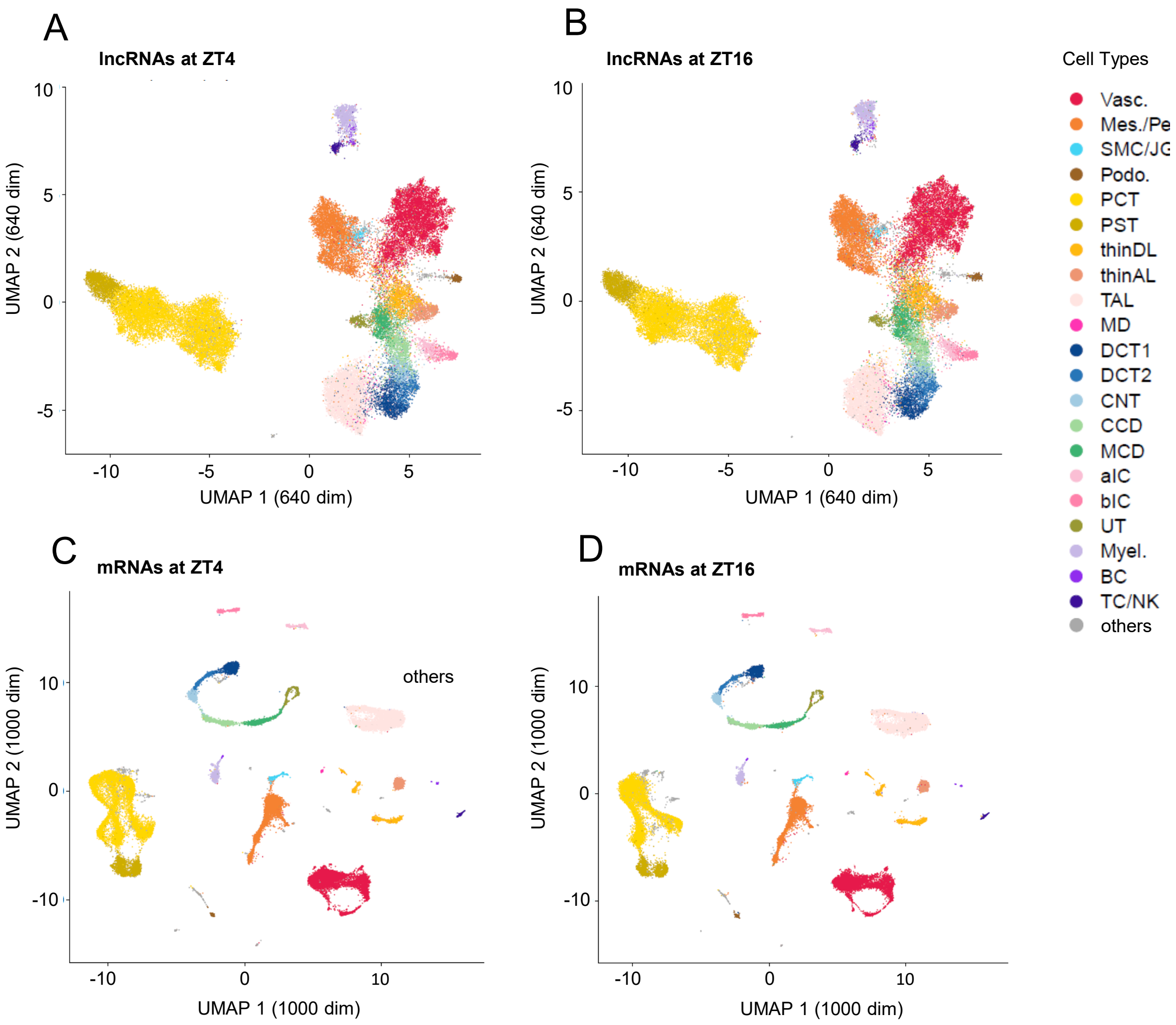

Figure S2: Cell types in UMAPs of lncRNAs and mRNAs, split by ZT

Figure S2: Cell types in UMAPs of lncRNAs and mRNAs, split by ZT. A–B. UMAP generated from renal lncRNAs in Ctrl and cKOt mice (*N*=8), then split by time point. Nuclei are colored by manually attributed cell type/category based on clustering analysis performed exclusively on mRNAs. Apart from coloring, UMAPs are the same as in Figures 3B and 3H. **C–D.** UMAP generated from renal mRNAs in Ctrl and cKOt mice (*N*=8), then split by time point. Nuclei are colored by manually attributed cell type/category based on clustering analysis performed exclusively on mRNAs. Apart from coloring, UMAPs are the same as in Figures 3E and 3K.

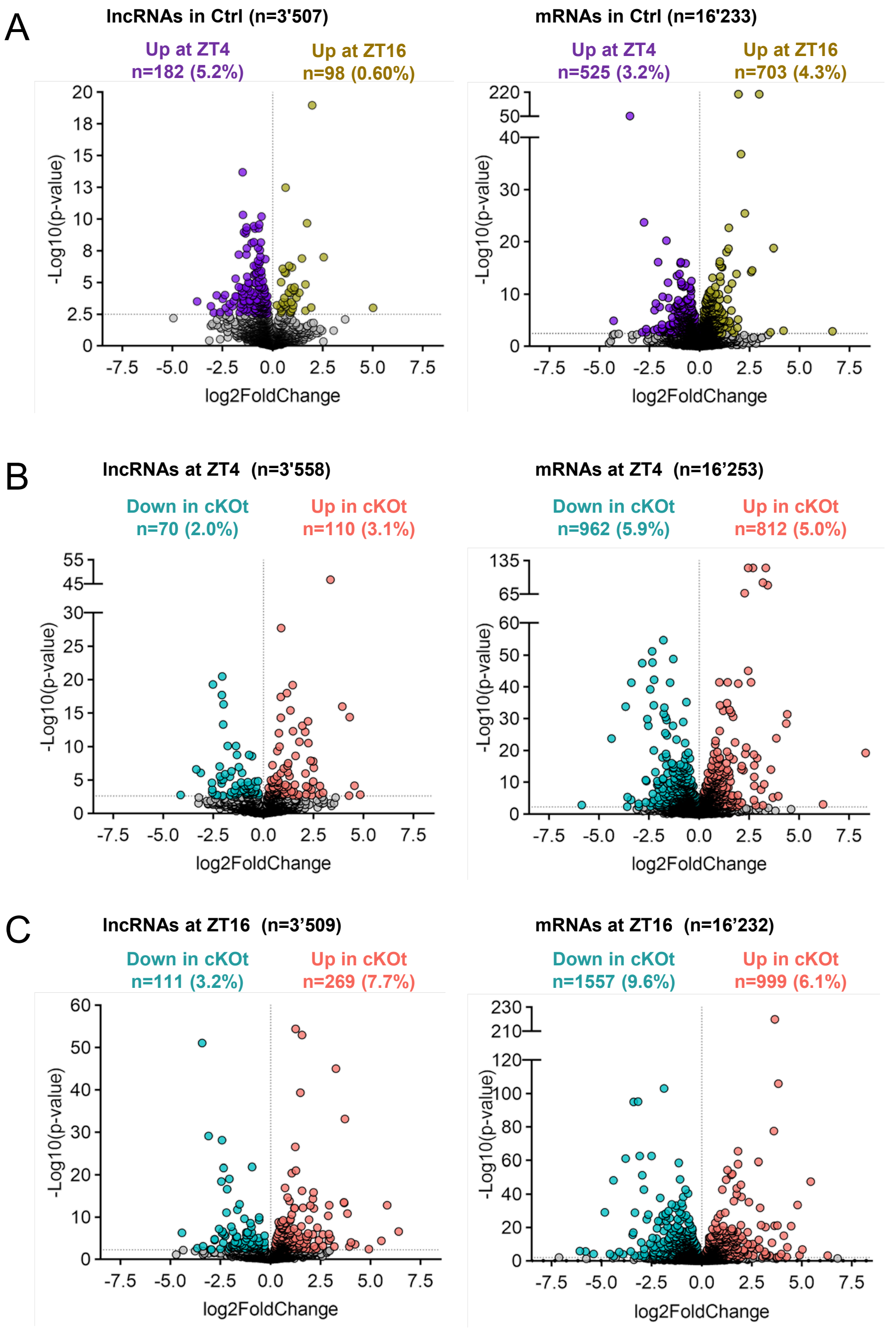

Figure S3: Effects of circadian time and the circadian clock on renal lncRNA and mRNA expression in deeply sequenced bulk RNA-seq data.

Figure S3: Effects of circadian time and the circadian clock on renal lncRNA and mRNA expression in deeply sequenced bulk RNA-seq data. A. Volcano plots of differential expression between ZT4 and ZT16 in Ctrl mice based on pseudo-bulk analysis of the single-nucleus (SN) dataset. Significant fold changes (adjusted $p < 0.05$, threshold shown as dotted line) are highlighted in purple (more abundant at ZT4) or yellow (more abundant at ZT16). Numbers and percentages of differentially expressed lncRNAs are indicated at the top of the plot. **B–C.** Volcano plots of differential expression between cKOt and Ctrl at ZT4 or ZT16, based on pseudo-bulk analysis of the single-nucleus (SN) dataset. Left: lncRNAs. Right: mRNAs. Significant fold changes (adjusted $p < 0.05$, threshold shown as dotted line) are highlighted in blue (less abundant in cKOt) or red (more abundant in cKOt). Numbers and percentages of differentially expressed lncRNAs are indicated at the top of the plot.

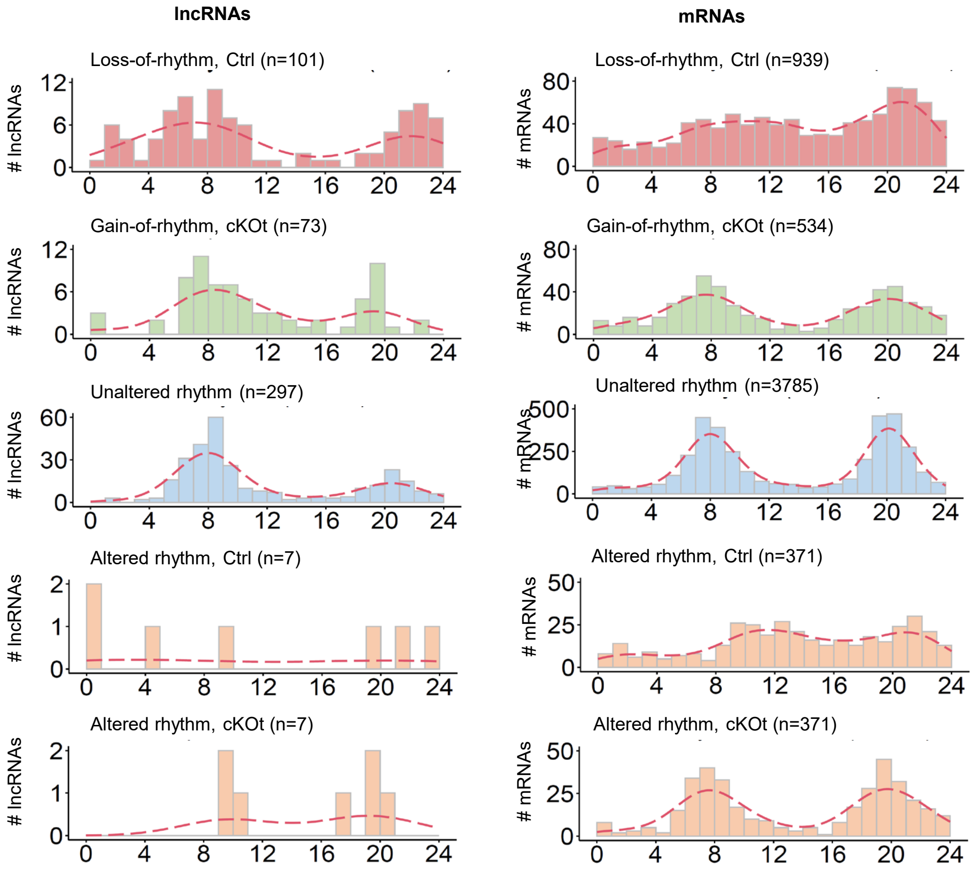

Figure S4: Acrophase distribution of renal lncRNAs and mRNAs per dryR rhymicity model.

Figure S4: Acrophase distribution of renal lncRNAs and mRNAs per dryR rhymicity model. Histogram showing the acrophase distribution of renal lncRNAs (left) or mRNAs (right) assigned to each of the dryR rhythmicity models 2, 3, 4, and 5. Red dashed lines: kernel density estimates.

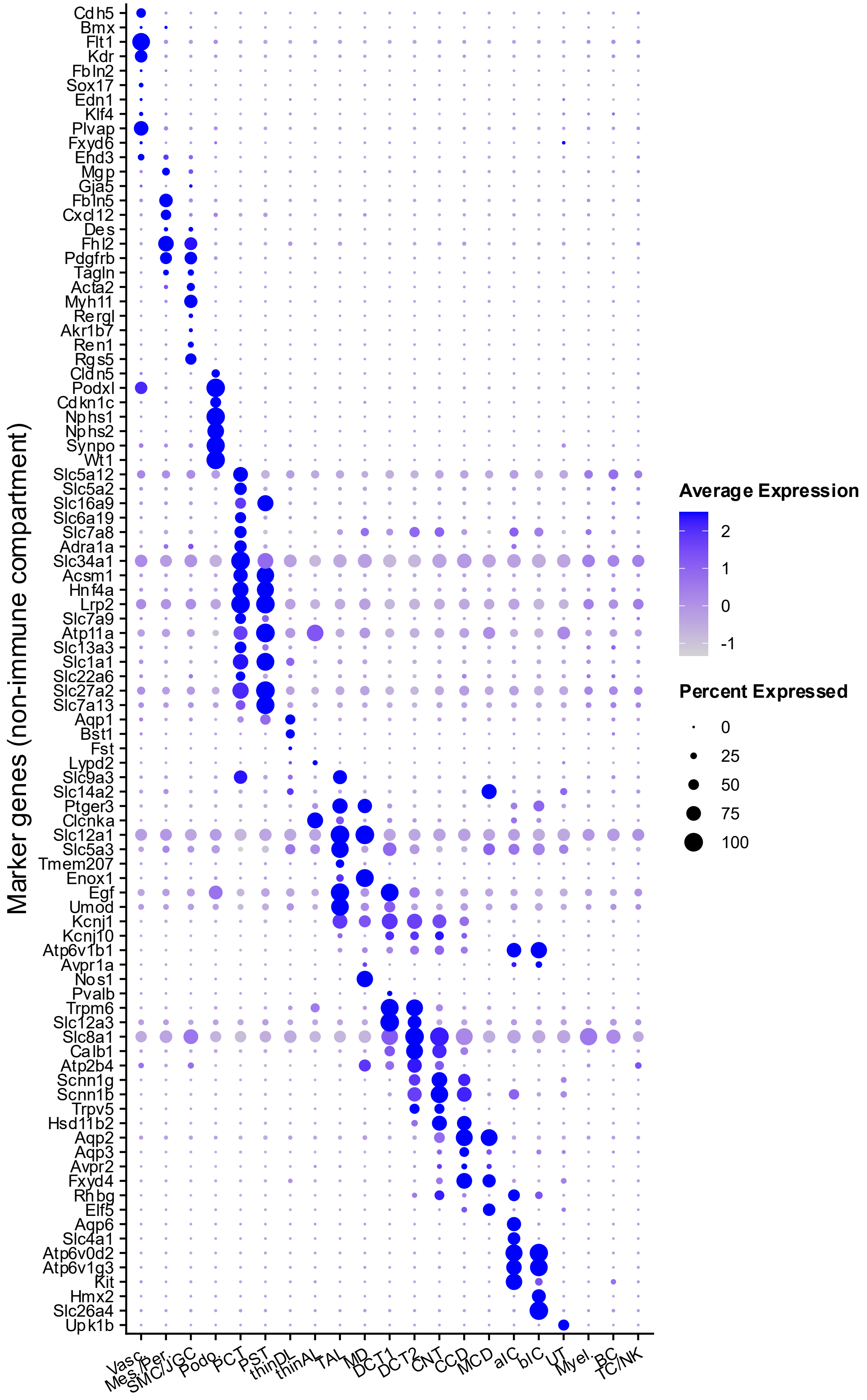

Figure S5: Renal marker genes delineating cell types of the non-immune compartment.

Figure S5: Renal marker genes delineating cell types of the non-immune compartment. Expression levels of marker genes by cell type, computed after manual assignment of cell clusters to cell types. Average expression (dot color): mean log-normalized expression per cell, with Seurat's default scaling. Percent expressed (dot size): Fraction of cells with non-zero expression. Plot generated using the Seurat R package with default parameters.

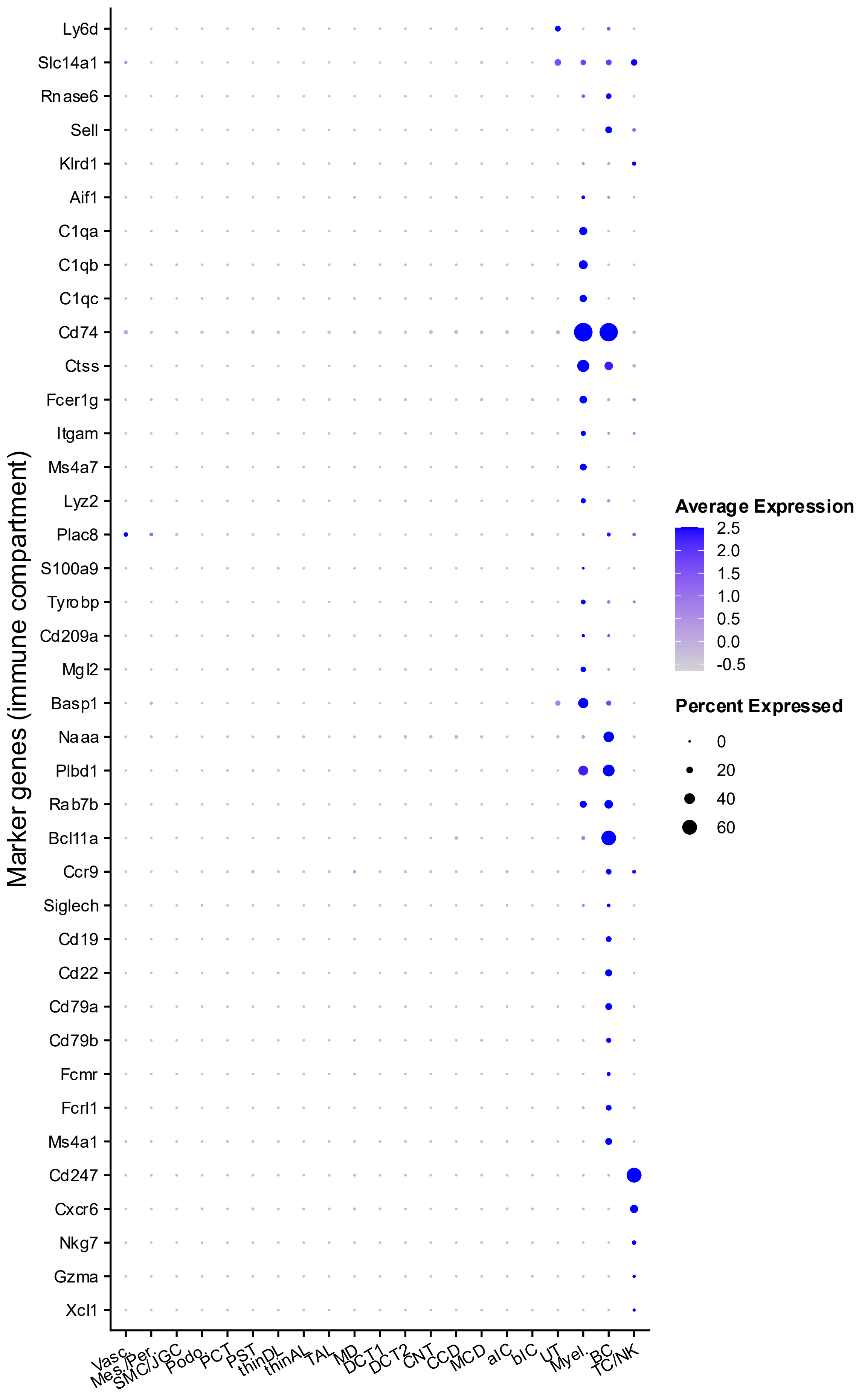

Figure S6: Renal marker genes delineating cell-type groups of the immune compartment.

Figure S6: Renal marker genes delineating cell type groups of the immune compartment.

Expression levels of marker genes by cell-type group of the immune compartment, computed after manual assignment of cell clusters to cell types. Average expression (dot color): mean log-normalized expression per cell, with Seurat's default scaling. Percent expressed (dot size): Fraction of cells with non-zero expression. Plot generated using the Seurat R package with default parameters.
